# A Literature Review of the Effects of Air Pollution on COVID-19 Health Outcomes Worldwide: Statistical Challenges and Data Visualization

**DOI:** 10.1146/annurev-publhealth-071521-120424

**Published:** 2022-12-21

**Authors:** A. Bhaskar, J. Chandra, H. Hashemi, K. Butler, L. Bennett, Jacqueline Cellini, Danielle Braun, Francesca Dominici

**Affiliations:** 1 Department of Government, Harvard University, Cambridge, Massachusetts, USA; 2 Harvard Medical School, Harvard University, Boston, Massachusetts, USA; 3 Environmental Systems Research Institute, Redlands, California, USA; 4 Countway Library of Medicine, Harvard Medical School, Harvard University, Boston, Massachusetts, USA; 5 Department of Biostatistics, Harvard T.H. Chan School of Public Health, Harvard University, Boston, Massachusetts, USA; email: fdominic@hsph.harvard.edu; 6 Department of Data Science, Dana-Farber Cancer Institute, Boston, Massachusetts, USA

**Keywords:** air pollution, COVID-19, data science, visualization, evidence synthesis

## Abstract

Several peer-reviewed papers and reviews have examined the relationship between exposure to air pollution and COVID-19 spread and severity. However, many of the existing reviews on this topic do not extensively present the statistical challenges associated with this field, do not provide comprehensive guidelines for future researchers, and review only the results of a relatively small number of papers. We reviewed 139 papers, 127 of which reported a statistically significant positive association between air pollution and adverse COVID-19 health outcomes. Here, we summarize the evidence, describe the statistical challenges, and make recommendations for future research. To summarize the 139 papers with data from geographical locations around the world, we also present an open-source data visualization tool that summarizes these studies and allows the research community to contribute evidence as new research papers are published.

## INTRODUCTION

On May 16, 2022, the Associated Press reported that the US death toll from COVID-19 hit 1 million. On March 23, 2022, the White House reported that the most common way that COVID-19 is transmitted from one person to another is through tiny airborne particles of the virus hanging in indoor air for minutes or hours after an infected person has been there (45). At the same time, several hundred papers have been published around the world, providing evidence of a potential link between exposure to air pollution [including fine particulate matter (PM_2.5_)] and an increased number of COVID-19 cases and deaths.

To support the scientific community in their work to mitigate risk and develop solutions to address the global COVID-19 crisis, investigators must evaluate the evidence and identify research opportunities on the potential associations between short- and long-term exposure to air pollution and COVID-19 health outcomes. Benmarhnia et al. (5) and Copat et al. (15) published the earliest reviews and discussed (*a*) potential biological mechanisms linking exposure to air pollution and COVID-19 outcomes; (*b*) how to clearly formulate causal questions of interest; and (*c*) considerations for using quasi-experimental designs. Subsequent literature reviews (1, 58, 65) were valuable but limited in scope. They often included no more than 20 studies and discussed primarily limitations related to the emerging literature at the beginning of the pandemic. They commonly focused on the need to consider socioeconomic confounding variables (1, 7, 15), additional routes of COVID-19 transmission (1, 65), and the underestimation of case and mortality data (15). Particular studies emphasized more unique and underdiscussed aspects of the literature, such as the relationship of COVID-19 to rainfall, humidity, and climate change (68), as well as geographical variations between high- and low-income countries (65). All of these were narrative literature reviews, which led to a more subjective evaluation of the results.

Villaneuve & Goldberg (58) included nine studies in their review and focused on the methods in these few articles. Their primary objective was to critique the methodological approaches used in these studies and to advocate for peer review. Most recently, two additional review articles (39, 68) have summarized the results of a greater number of studies (51 and 68 articles, respectively). While these papers synthesized information about the state of the evidence, they omitted a deep dive into the statistical challenges and any quantitative review of the evidence.

This article offers a novel contribution to this expanse of research in a variety of ways. This review encompasses 139 articles, which substantially surpasses previous reviews in the scope of the included literature. Furthermore, this number of articles is important because it allows us to make more representative evaluations of the statistical challenges as well as opportunities that remain to improve the understanding of the association between air pollution and COVID-19.

In this article, we aim to achieve three goals: (*a*) summarize the evidence regarding the association between exposure to air pollution and COVID-19 outcomes; (*b*) provide a deep-dive discussion of the main statistical challenges and shortcomings in existing work, which both prompt areas of future research; and (*c*) introduce a data visualization tool for summarizing the evidence in an interactive, dynamic, and accessible manner.

## METHODS

The following two sections outline the methods by which we comprehensively searched the existing literature for studies on the effects of air pollution on COVID-19 spread and severity. We provide the search terms and databases used, in addition to the screening process and criteria for study selection to improve reproducibility. Finally, we describe the development of the online dashboard to visualize the results of the literature review.

### Search Strategy

We conducted a literature search of the National Library of Medicine’s PubMed database (https://pubmed.ncbi.nlm.nih.gov/), Elsevier’s Embase database, Clarivate Analytics Web of Science database, Cochrane’s COVID-19 Study Register, and Cold Spring Harbor Laboratory’s medRxiv and bioRxiv databases. The full search queries used for each database are provided in Supplemental Table 1.

### Study Eligibility Criteria and Study Selection

**Figure 1:**
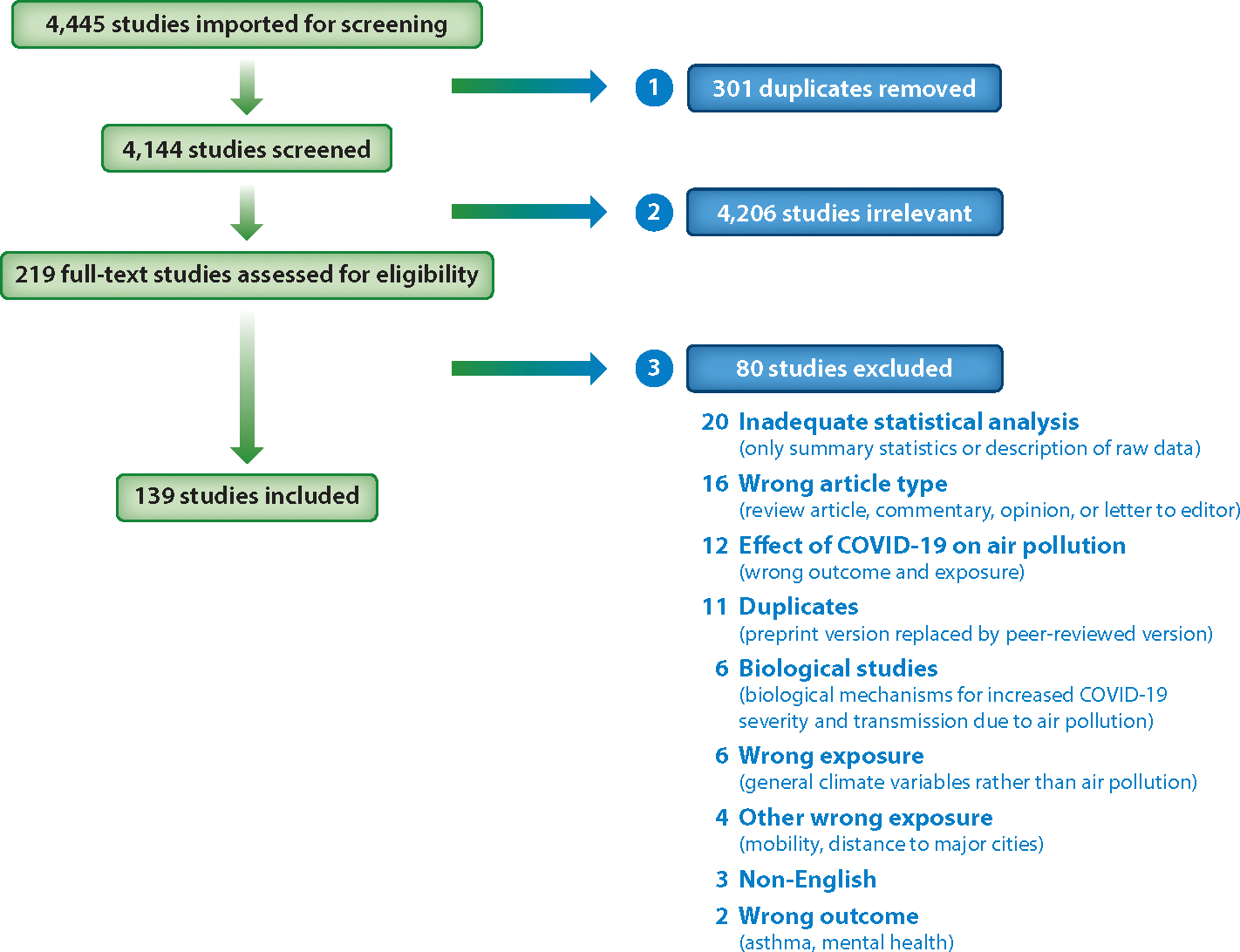
PRISMA figure displaying the screening process for the 4,445 studies identified in the database searches run on May 21, 2021. In the first round, duplicate studies were removed. In the second round, studies deemed irrelevant by title and abstract review were removed. Finally, studies deemed irrelevant by full-text review were removed, resulting in a total of 139 studies included in the review.

Inclusion/exclusion criteria were designed to identify original research describing the effects of air pollution on COVID-19 health outcomes (Figure 1). We included both peer-reviewed and preprint studies due to the quickly evolving nature of the topic. Although preprint studies are preliminary, we decided to include them because they provide useful information regarding statistical approaches and can inform future research directions. Only studies written in English and published online up to May 21, 2021, were included. We limited our evidence synthesis to articles that relied on data prior to widespread availability of the COVID-19 vaccine ( January 2021), as vaccination had a significant effect on the trajectory of COVID-19 health outcomes.

Two screeners reviewed each identified paper from the database searches using the Covidence online platform (https://www.covidence.org/). Duplicate papers were removed. The studies were then put through a coarse-grain selection process in which studies were excluded based on a review of the title and abstract. Papers that did not discuss air pollution and COVID-19 health outcomes were excluded. In the second round, full texts were reviewed. Studies that discussed the effects of short- or long-term exposure to air pollution on COVID-19 health outcomes were included. Studies were not restricted based on the temporal and spatial resolution of air pollution data and COVID-19 health data and also not restricted on the basis of COVID-19 health outcomes or confounder selections.

Studies investigating the effects of COVID-19 and the lockdowns caused by COVID-19 on air pollution were excluded. In addition, many studies published early in the pandemic described the relationship between COVID-19 cases and air pollution without significant quantitative or statistical analysis. These studies concluded that air pollution is related to coronavirus spread because early cases in the pandemic were concentrated in the highly polluted Po Valley and other highly polluted areas of China (23, 40). These studies, which provided only cursory analysis, were also excluded.

### Visualization and Automation Methodology

The process of spatially and temporally summarizing and visualizing the results of this comprehensive literature review was automated using Python version 3.9.11 running in a Jupyter Notebook environment. This approach created an environment where researchers can easily reuse or customize the automation of the visualization and evidence synthesis process presented in this article.

We created a spreadsheet that contained the following features (manually annotated) that summarized relevant attributes of each article: digital object identifier (DOI), type of study (long-term, short-term time series, short-term cross-sectional), geographical area of study (country or countries examined), areal unit (smallest area/region type studied in each paper), outcome (cases, deaths, hospitalizations, etc.), statistical method (correlation, linear regression, mixed models, etc.), statistical significance (statistical significance was recorded for each air pollutant studied), covariates (covariates controlled for in the final model), and code availability for each paper (e.g., code made publicly available on GitHub). The DOI was used to access bibliographic databases to retrieve additional information on each article (e.g., number of citations). The study area information was used to georeference each article. These attributes allowed end users of the evidence synthesis visualization tool to filter and select specific groups of articles based on their attributes. This spreadsheet was also critical for determining statistical challenges and summarizing each paper for this review.

The spreadsheet was read using Python (see Supplemental Table 2), which then launched an automated workflow to generate a series of maps and Web applications that synthesize the articles using the following process. First, each article’s metadata is extracted from the Crossref metadata repository (https://www.crossref.org/) using the DOI provided. This search returns a properly formatted citation with the article, publication year, author names, and citation count. Next, each article’s annotated study area is geocoded using a proprietary Python library, ArcGIS API for Python (https://developers.arcgis.com/python/). The data extracted from Crossref, the geocoded data from the ArcGIS API for Python, and the previously mentioned manually entered article are together used to produce or update a series of information products that synthesize the collection of articles provided by the researcher (Table 1).

## RESULTS

Here, we provide a summary of the articles selected after the literature review search and application of the exclusion/inclusion criteria. In particular, we focus on the study types, location of the studies, outcomes, exposures, statistical models used, overall findings, and covariates controlled for. Finally, we describe the visualization of the results from this literature review through the online dashboard and the process by which researchers can submit new articles for inclusion in the dashboard.

### Selected Studies

**Figure 2:**
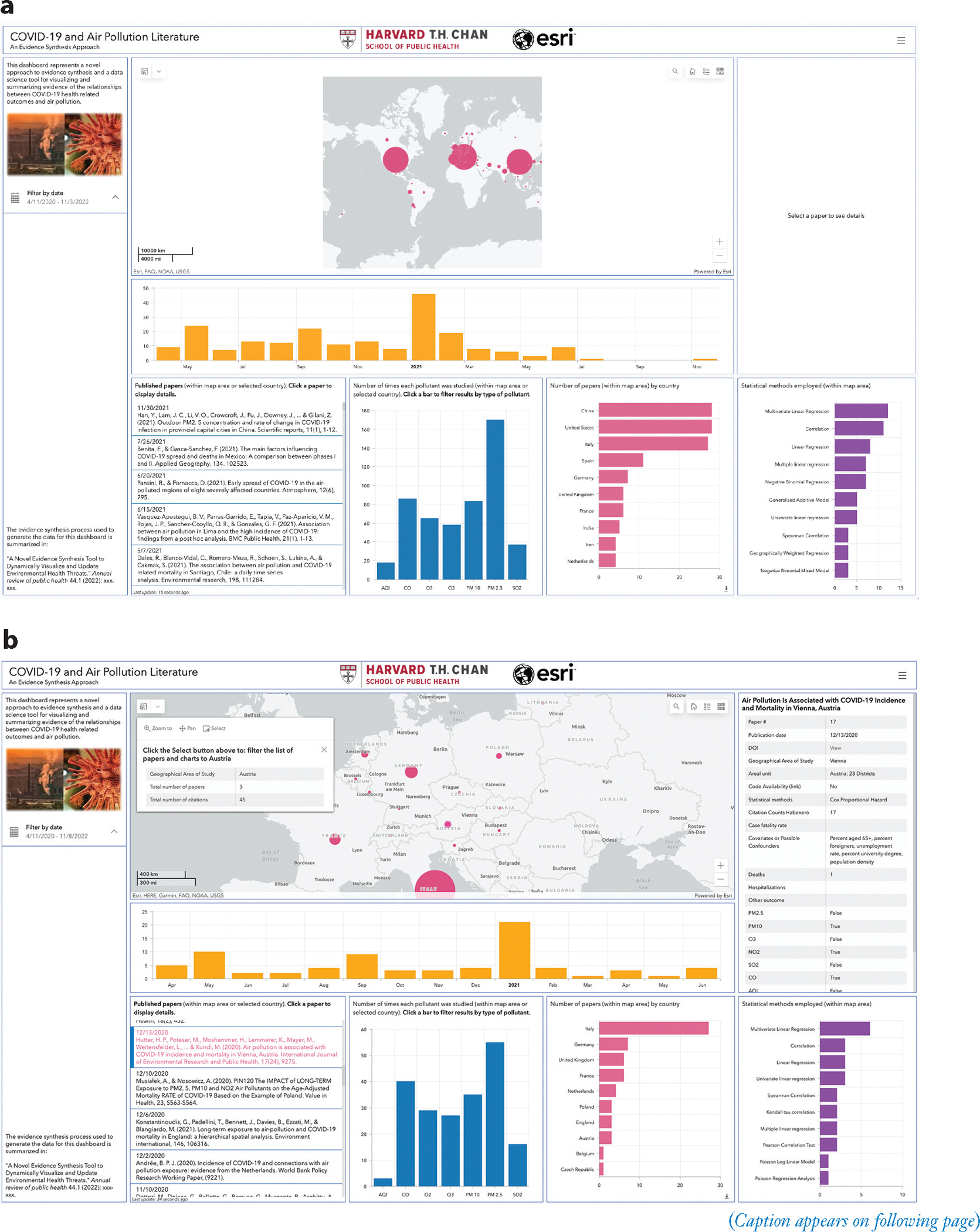
(*Figure appears on preceding page*) Screenshots of the evidence synthesis tool dashboard (http://bit.ly/3hzcsbv). (*a*) Zoomed out dashboard containing information from most countries, including the three most studied countries (Italy, China, United States). (*b*) Zoomed into Central Europe. The air pollutant types, papers within each country, and statistical methods chart have changed accordingly. In addition, the Austria graduated circle and a specific paper from Austria have been selected and the associated text is displayed. The short video demonstrating the capabilities of the dashboard is available at https://vimeo.com/709361095.

The search yielded 4,144 articles across all databases searched (Figure 1). After excluding research studies that did not discuss both air pollution and COVID-19 (based on abstract review), 219 articles were eligible for full-text review. Of these, 139 were research studies published in English that investigated the effects of air pollution on COVID-19 health outcomes (based on full-text review). Only 8 of the 139 articles included open access to their code. Of these 139 articles, 134 were peer-reviewed. These results can be visualized in the interactive database described above (Figure 2; http://bit.ly/3hzcsbv).

### Study Types

The results were summarized by the type of air pollution effect being examined. Studies focusing on long-term effects of air pollution (63 papers) were grouped together. These studies examined the impact of air pollution exposure over a long period of time prior to the severe acute respiratory syndrome coronavirus 2 (SARS-CoV-2) outbreak. The underlying hypothesis of these studies is that exposure to air pollution over long periods of time negatively impacts respiratory health, thereby increasing susceptibility to SARS-CoV-2 infection and more severe COVID-19 health outcomes. All these studies relied on a cross-sectional study design.

Studies focusing on the short-term effects of air pollution on COVID-19 health outcomes (61 papers) were grouped together. These studies compared day-to-day variations in air pollution with day-to-day variations in COVID-19 health outcomes. Studies focusing on cross-sectional analyses of the short-term effects of air pollution on COVID-19 health outcomes (15 papers) were grouped together. These studies compared air pollution levels during the outbreak or shortly before the outbreak with COVID-19 health outcomes. The underlying hypothesis of both the time series and cross-sectional studies is that recent air pollution exposure increases the transmission of the virus or the severity of COVID-19.

### Location of the Studies

The 139 studies examined the effects of air pollution on COVID-19 health outcomes across many different countries, primarily in North America, Europe, and Asia. Many of the studies focused on cities and provinces in China (32 papers), regions and provinces in Italy (32 papers), and counties in the United States (29 papers), all regions where the SARS-CoV-2 outbreak occurred early and was prominent. Other papers examined countries that were less prominent in the early part of the pandemic such as Spain (14 papers), United Kingdom (11 papers), Germany (9 papers), India (6 papers), Austria (5 papers), the Netherlands (5 papers), South Korea (4 papers), Mexico (4 papers), Poland (4 papers), Pakistan (3 papers), Bangladesh (3 papers), Finland (3 papers), and Peru (3 papers).

### COVID-19 Health Outcomes and Air Pollution Exposures

Five different COVID-19 health outcomes were reported across the 139 studies: cases, deaths, reproductive ratio, case fatality rate, and hospitalizations. The most frequently used health outcomes among both short-term and long-term studies were the number of COVID-19 cases (95 papers) and the number of COVID-19 deaths (65 papers) followed by case fatality rate (13 papers), hospitalizations (8 papers), and reproductive ratio (4 papers). Supplemental Table 2 provides additional information about the health outcomes utilized across these studies. The air pollutants most frequently studied across the 139 studies were PM_2.5_ (107 papers), PM_10_ (67 papers), O_3_ (53 papers), NO_2_ (67 papers), SO_2_ (31 papers), and CO (33 papers). In addition, 18 papers included the Air Quality Index (AQI) and a few papers studied aerosols, NH_3_, and formaldehyde (HCHO). Note that many papers studied more than one pollutant.

### Models

A wide diversity of modeling techniques was used in the 139 papers to make inferences about the relationship between air pollution and COVID-19 health outcomes. The most frequently used technique among both short-term and long-term studies was linear regression (32 papers) followed by Spearman and Kendall tau correlation estimation (30 papers), negative binomial regression (18 papers), generalized additive models (10 papers), Poisson regression (15 papers), mixed models (13 papers), and geographically weighted regression (4 papers). Recent studies have used other, more complex techniques such as Bayesian modeling (3 papers) and artificial neural networks or machine learning (3 papers). See Supplemental Table 2 for more specific information on statistical modeling.

### Statistically Significant Findings

**Figure 3:**
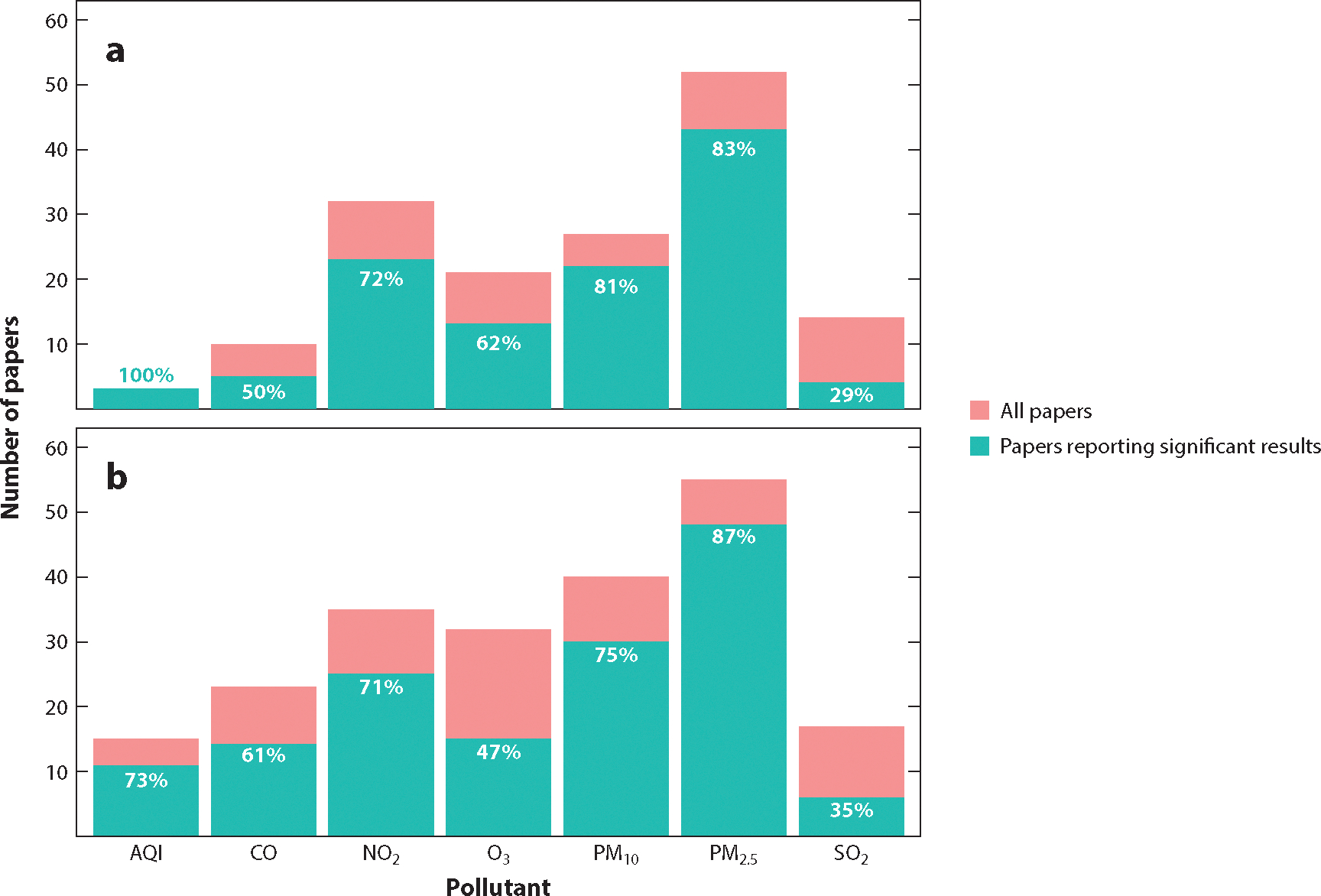
Evidence synthesis results stratified by the type of air pollutant studied. There were a total of 139 studies (76 short-term, 63 long-term), and many studies studied multiple pollutants. The total number of studies (*blue-green* and *pink*), number of studies that reported a statistically significant positive association between the air pollutant and COVID-19 outcomes (*blue-green*), and percent of papers that report significant results (*white numbers*) are presented for each pollutant. (*a*) Long-term studies (AQI, *n* = 3; CO, *n* = 10; NO_2_, *n* = 32; O_3_, *n* = 21; PM_10_, *n* = 27; PM_2.5_, *n* = 52; SO_2_, *n* = 14). (*b*) Short-term studies (AQI, *n* = 15; CO, *n* = 23; NO_2_, *n* = 35; O_3_, *n* = 32; PM_10_, *n* = 40; PM_2.5_, *n* = 55; SO_2_, *n* = 17).

Of the 139 studies, 127 reported a statistically significant positive association between air pollution and adverse COVID-19 health outcomes (Figure 3). Fifty-eight of the 63 papers that studied the long-term effects of air pollution found a statistically significant relationship with COVID-19 outcomes. Sixty-nine of the 76 papers that studied the short-term effects of air pollution found a statistically significant relationship with COVID-19 outcomes. For both short-term and long-term papers, a significant association between PM_2.5_ and COVID-19 outcomes was reported in the highest proportion of papers (87% and 83%, respectively). PM_10_ and NO_2_ associations with COVID-19 were reported in the next highest proportion of both study types, followed by O_3_ and CO. A low proportion of papers reported statistically significant relationships between SO_2_ and COVID-19 outcomes for both short-term (35%) and long-term (29%) effects. AQI was significantly associated with COVID-19 outcomes in all long-term studies and 73% of the short-term studies.

### Covariates

The variables that were adjusted for substantially differed between the papers. By design, the 30 articles that used correlation did not adjust for any variables. The short-term time series articles tended to adjust for temperature, wind speed, humidity, precipitation, air pressure, and day of the week. Some articles did not adjust for any confounders, but a majority of the articles adjusted for some confounders. Variables that were adjusted for in the long-term and short-term cross-sectional studies varied the most. Fifty-one of the 57 cross-sectional articles that considered covariates, at the very least, adjusted for age structure, gender, income, race, or education. In other words, sociodemographic factors were generally well-controlled for; however, inclusion of other covariates varied substantially. A large proportion of papers (22 out of 57 papers) controlled for health care system capacity through variables such as number of hospital beds, number of physicians, number of COVID-19 tests available, etc. Many articles (27 out of 57 papers) also controlled for the overall health status of the people by including, for example, the proportion of smokers and the proportion of people with known comorbidities (obesity, diabetes, chronic obstructive pulmonary disease, asthma, etc.). Seven out of the 60 articles controlled for the first case identified in the area (timing of the epidemic curve) and 5 of the 60 articles controlled for mobility/movement in the region. See Supplemental Table 2 for the specific covariates accounted for in each study.

### Visualization of the Evidence

In this section, we describe our data visualization tool of evidence synthesis. Our open science approach was carried from creation of the database through visualization and evidence synthesis. Table 1 summarizes the types of information products produced by the evidence synthesis. A screenshot of the interactive dashboard (http://bit.ly/3hzcsbv) is shown in Figure 2 and is divided into several panels. A short video demonstrating the capabilities of the dashboard is available at https://vimeo.com/709361095.

The center panel of the dashboard shows a map depicting where the relationships between health outcomes and air pollution have been studied. The circle size represents the number of papers from studies that considered data from a particular country. Clicking on the graduated circle for a country displays a summary of the number of papers and the total number of citations for those papers. Panning and zooming the map allow researchers to focus on a particular region. The summary charts of total papers by country, papers by time, pollutants investigated, and statistical methods employed in the bottom panels of the dashboard change as the map changes. The leftmost panel provides background information on the dashboard and allows researchers to filter papers by publication date. Finally, the rightmost panel displays detailed information on a selected paper, including a DOI link.

**Figure 4:**
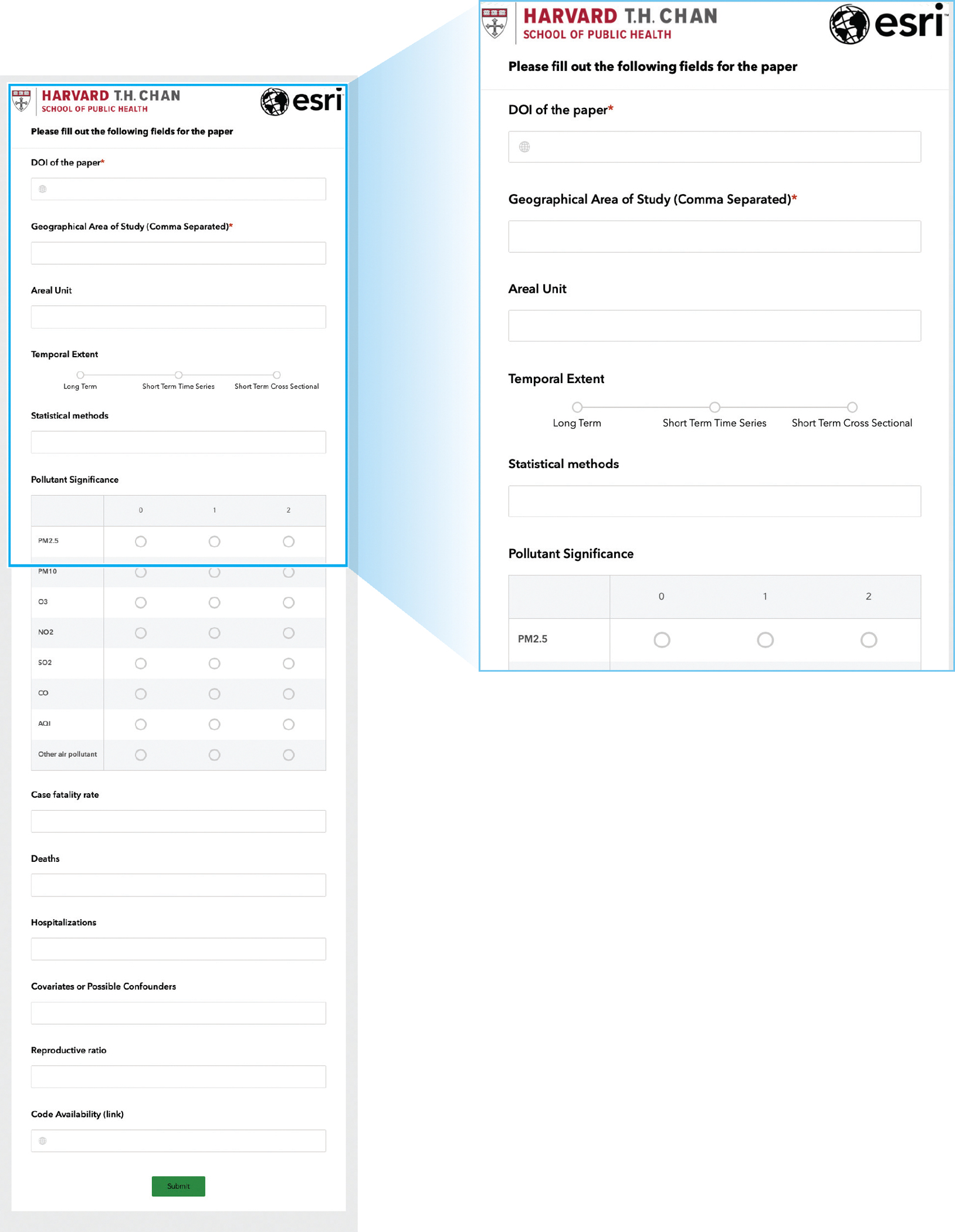
Screenshots of the fields in Survey123 (https://bit.ly/3hGdIti).

An important feature of this tool is that it allows other researchers to upload more recent papers. We created a Web-based application (https://survey123.arcgis.com) that enables researchers to provide new data entries (new published papers) to be added to the dashboard. Researchers need to fill out two required fields to submit a new paper: the DOI of the paper and the geographical area of the study. Other fields in the form are optional; Figure 4 depicts the complete list of fields in the survey (as a screenshot). We highly recommend that researchers fill out as many fields as possible to maintain the integrity and comprehensive nature of the database. After submitting a new paper, the entry will be checked for data sanity. If the entry passes the sanity check, the dashboard will be modified automatically.

## DISCUSSION

In this article, we have reviewed the literature for air pollution effects on COVID-19 health, summarized the evidence, and outlined the statistical methods used and challenges. Furthermore, we describe our development of an open-source data visualization tool to overcome the difficulty of synthesizing evidence from a large number of studies with heterogeneous study designs and statistical approaches from around the world. Overall, we found that 127 out of 139 papers reported a statistically significant positive association between air pollution and adverse COVID-19 health outcomes (Figure 3). However, many statistical challenges affect the validity of these studies, as summarized below.

### Methodological Challenges in Epidemiological Studies of Air Pollution and COVID-19

Understanding the relationship between short- and long-term exposure to air pollution and adverse COVID-19 health outcomes is crucial for developing solutions to this global crisis. In determining the strength of the evidence regarding the association between air pollution and its adverse effects on COVID-19 health outcomes, some well-known challenges in air pollution epidemiology become even more serious, and several additional complications emerge. These new challenges are mostly related to the reality that the data on COVID-19 are imprecise, are constantly evolving, come from a variety of sources, and are primarily available only at the aggregate level (e.g., county, regional, or country level). Indeed, the quality of the data and the lack of available well-validated electronic health record data at the national level (particularly in the United States) render the general challenges in evaluating causality even more difficult to address. Villeneuve & Goldberg (58) pointed out similar methodological challenges and advocated for a peer review of these studies. We agree with their assessment and discuss these issues below through a more optimistic lens by highlighting research opportunities that could increase the scientific rigor of these studies.

#### Data quality.

The validity of the COVID-19 health outcomes data is questionable, as there is no uniform case definition of a COVID-19 death and diagnostic errors are made in COVID-19 cases (24). This lack of validity in COVID-19 health outcomes data could contribute to a high degree of over- or underreporting. Many infected people may have died without ever having been tested for SARS-CoV-2 infection; whereas in other cases, COVID-19 might have been secondary to the cause of an individual’s death. Even papers from the same location used data from multiple sources with different definitions of COVID-19 cases and deaths, which contributes to the variability in study results (9, 71). Alternatively, excess mortality is an outcome type that has been used in natural disasters and outbreaks to account for the difficulties in identifying cause-specific mortality. One of the studies used excess mortality as their outcome of interest (17). In addition, in the early phases of the pandemic, and even currently, SARS-CoV-2 testing was not universally available due to shortages in test kits and the relatively late adoption of mass-scale testing. Therefore, only a subset of the population has been tested, and we cannot account for those who are asymptomatic but infected. While outcome misclassification is of great concern, to actually bias the analyses, outcome misclassification would need to vary in both space and time as the exposure varies. This potential bias is possible to assess using validation data that diagnose COVID-19 accurately. In time series studies, to bias the analysis, the outcome misclassification will need to vary daily and be correlated with daily variation in air pollution. Thus, access to a nationwide registry with validated cause of death and hospitalization data is highly desirable. While they are far from ideal, specific data sets from China and Germany do provide standardization in the COVID-19 case and mortality definitions according to national laws (30, 62). These examples stand in contrast to the more heterogeneous reporting in the United States.

#### Ecological fallacy.

Only 5 of the 139 studies in our case study were individual-level studies as opposed to ecological studies (8, 10, 38, 50, 57). Ecological fallacy is a formal fallacy in the interpretation of statistical data that occurs when inferences about the nature of individuals are deduced from inferences about the group to which those individuals belong [see for example Jackson et al. (31)]. While ecological studies are useful in generating preliminary evidence in data-scarce settings, increasing the scientific rigor of research in this area will require access to nationally representative, individual-level data on adverse COVID-19 health outcomes, including information about patients’ residential addresses, demographics, and individual-level confounders. More data at the individual level are becoming available, but only for subsets of the population. With these data, it will be possible to leverage the extensive literature to augment aggregate data with individual-level data and adjust for ecological bias (35, 59). These types of studies could substantially increase the credibility of the results; however, developing a national and publicly available registry on individual-level data on COVID-19 patients is an enormous challenge that will require many privacy, legal, and ethical trade-offs (54).

#### Other determinants of COVID-19.

Ecological data typically do not allow for adjustment by individual risk factors such as age, gender, ethnicity, or occupation. Age is one of the strongest predictors of survival for most conditions, including COVID-19. Gender-based differences in time-activity patterns contribute to different levels of air pollution exposure between men and women, and women have been shown to be more susceptible to several environmental exposures (13). Occupation has also been an important factor in the COVID-19 pandemic; those who provide medical care as well as other essential workers, such as those working in meat-packing plants, are at increased risk of developing SARS-CoV-2 infection (44). These challenges can be overcome by having access to some individual-level data that provide this additional information. Each of the five articles in this review that had access to individual data controlled for some but not all these factors. In particular, occupation was not explicitly considered except for a binary variable that indicated whether an individual was working from home or not (38).

#### Accounting for differences in socioeconomic status.

Disadvantaged people (e.g., those without health insurance, those who are undernourished, and those with poorly managed underlying health conditions, such as cardiovascular conditions and/or diabetes) have a greater susceptibility for both contracting SARS-CoV-2 and dying from COVID-19. Myriad social and economic factors contribute to high rates of infection and put individuals at higher risk from the sequelae of COVID-19, and disparities in COVID-19–related outcomes may be due to social deprivation rooted in long-standing racial and socioeconomic inequities. This issue has been raised by a number of authors [see for example Chowkwanyun & Reed (12) and Yancy (63)]. Differences in socioeconomic status do not vary daily, so they will not affect the results of time series analyses; however, they are very important confounders in long-term effect studies of air pollution exposure and are potential effect modifiers in both short- and long-term effect studies. Thirty-six of the 78 cross-sectional studies considered these factors. Even without individual-level covariates on socioeconomic status, it is possible to conduct studies stratified by subsets of geographical location that have certain characteristics (such as a high percentage of residents in poverty or a high percentage Black population). These studies could provide important insights into the factors that increase vulnerability to adverse COVID-19 health outcomes due to short- or long-term exposure to air pollution. None of the 139 studies used this stratification approach.

#### Granularity of data.

It is difficult to gather individual-level data especially in an emerging pandemic. If data need to be aggregated to a unit of area, smaller areas are preferable to larger ones. Fine variations in confounding variables and air pollution concentrations can drastically vary if the unit of area is very large. The most common unit of area used in the studies reviewed were counties in the United States (15 out of 29 papers), provinces in Italy (14 out of 32 papers), and cities or provinces in China (26 out of 32 papers). The largest county in the United States has ~10 million people, the largest province in Italy has ~4 million people, and the largest province in China has ~126 million people. It is clear that grouping this many people into a single data point will reduce accuracy of the estimates. A few of the reviewed papers attempted to combat this issue by using very small units of area. Coker et al. (14) used Italian municipalities, which are much smaller than Italian provinces (7,914 municipalities versus 110 provinces). Konstantinoudis et al. (34) used English Lower Layer Super Output Areas (LSOAs) rather than the larger local authority level used in similar papers (333 local authorities versus 32,844 LSOAs). Future studies in the United States should follow this approach and attempt to use zip code–level data (nearly 42,000 zip codes versus 3,100 counties) to improve their analyses, especially as no cross-sectional study included in this review used a more granular unit of area than county.

#### Exposure error.

In all the studies included in our evidence synthesis, except for a few of the individual-level studies where home location was collected (8, 10, 50, 57), the same level of air pollution exposure was assigned to everyone living in large geographical areas. Therefore, spatial differences in exposure were not captured. Several statistical approaches have been developed to propagate the different sources of uncertainty associated with exposure error into the statistical model to estimate health effects [see for example Dominici et al. (19), Gryparis et al. (25), and Szpiro et al. (56)]. These approaches have not yet been implemented in studies of air pollution exposure and adverse COVID-19 health outcomes and represent an exciting area of future research.

#### Physical distancing and timing of the epidemic curve.

The implementation of public health policies, which vary widely by jurisdiction, has been successful in reducing SARS-CoV-2 transmission and flattening the epidemic curve. For example, in Georgia, areas that did not adopt physical distancing practices experienced higher incidence and mortality from COVID-19 when compared with other areas in the state that did practice physical distancing. Cities in California that tend to have higher levels of fine particulate matter adopted stay-at-home policies earlier than did other regions. Because these policies differ by regional air pollution levels, including rural and urban areas within the same county, they can distort the observed associations between air pollution and adverse COVID-19 health outcomes. In order to partially account for this distortion, a few studies used hierarchical/mixed-effects models with random effects at the geographic unit where public health policy is made (18, 21, 28, 36, 37, 42, 47, 51, 61).

There will be temporal differences in the number of COVID-19 cases and deaths by region. US counties were at very different stages on the epidemic curve, especially in early April 2020. Larger cities are more populous and tend to have increased travel to and from international locations, providing increased opportunity for the spread of COVID-19 early in the pandemic. These larger cities also tend to have higher concentrations of air pollution. In the context of cross-sectional analyses, there will be a greater number of cases and deaths in those cities that are further along on the epidemic curve, which further confounds analysis. Physical distancing and the timing of the epidemic curve are important potential confounders, especially for long-term studies and cross-sectional studies. Measuring these factors accurately while COVID-19 data are still accumulating is very challenging; however, several approaches can be implemented. For example, it is possible to conduct causal inference analyses using proxies for physical distancing and the stage of the epidemic as measured confounders in the regression analyses or included as covariates in propensity score analyses to ultimately compare geographical locations that are adjusted or matched with respect to these variables. Seven papers addressed timing on the epidemic curve by controlling for the first case (20, 49, 55, 61) and 5 papers partially addressed physical distancing by controlling for mobility (22, 26, 53, 60). These analyses can be informative, especially if augmented by sensitivity analyses to unmeasured confounding bias, such as the E-value (27). The E-value is defined as the minimum strength of association on the risk ratio scale that an unmeasured confounder would need to have with both the exposure and the outcome to fully explain away a specific exposure–outcome association, conditional on the measured covariates. Statistical software for E-value implementation is available (41). As more COVID-19 data (unfortunately) accumulate, these statistical analyses must be repeated routinely to assess the stability of the results with respect to the different phases of the pandemic and other potential unmeasured confounders. None of the articles calculated the E-value nor did any provide an in-depth analysis of unmeasured confounders.

#### Clustering of cases and deaths.

Unlike studies of long-term exposure to air pollution and chronic diseases where deaths can reasonably be assumed to be independent across individuals, both COVID-19 cases and COVID-19 deaths tend to occur in clusters. Clustering of cases and deaths has been widely reported, such as the now famous choir practice during which a large portion of attendees became ill or the tragic events in congregant settings such as retirement homes and long-term care facilities. Although some of the selected studies’ authors included random effects to account for clustering (18, 21, 28, 36, 37, 42, 47, 51, 61), without individual-level data, it is simply not possible to account for this clustering.

#### Lag time.

A key consideration in short-term time series studies is the lag time between the time series of COVID-19 outcomes and the air pollution time series. Across these 139 articles, 4 overall approaches were used: (*a*) choose a single biologically supported lag time such as 11 days for positive COVID-19 tests (cases) (3) and 18 days for deaths (33); (*b*) fit a different model for each single lag time (60); (*c*) fit a different model for each range of cumulative lags that is either the average or sum of pollutant concentration over *x* days before the given COVID-19 outcome value (46, 60, 67, 70); (*d*) use a distributed lag in which each lag concentration is given a distinct coefficient (16, 52); or (*e*) do not include a lag (2, 4, 6). We note the issue with multiple comparisons for statistical significance when considering many different lag ranges and recommend using either values that are in a biologically plausible range (6–12 days for cases and 2–3 weeks for deaths) or distributed lags where the maximum lag is the biologically plausible one. Both cumulative lags and single lags are acceptable in trying to determine a relationship between COVID-19 and air pollution; however, researchers should be aware that they are testing different hypotheses. When using single lags, the hypothesis is that air pollution is involved in carrying or transporting those viral particles. Cumulative lags account for multiple hypotheses. One hypothesis is the same as for the single lag. The other is that air pollution exposure once the virus has entered the body increases the severity of the disease, which leads to a greater number of cases, hospitalizations, and deaths.

#### Panel data.

Another consideration for short-term time series is how to combine estimates across different cities/regions. A few different approaches have been used thus far: (*a*) do a separate estimation of coefficients for each areal unit (4, 6); (*b*) use all the data in one large regression (29); (*c*) same as method *b* except for the use of cluster robust standard errors (67); (*d*) include fixed effects for each county (30, 69); (*e*) perform a meta-analysis with random effects (60, 64); and ( *f* ) use geographically weighted regression (46). Methods *b*, *c*, and *d* do not account for the fact that the short-term relationship between air pollution and COVID-19 outcomes can vary by location. Instead, a single number represents the relationship. Methods *a*, *e*, and *f* allow for the short-term relationship between air pollution and COVID-19 outcomes to vary by location. Method *a* is an extreme version of this approach whereby each location is given its own slope by running separate regressions. Method *d* operates under the assumption that the true relationship for each location is drawn from an overall distribution. It partially pools the estimates so that estimates from locations with small population sizes will be pulled toward the overall mean. Geographically weighted regression in this context will have estimates that are influenced by relationships in surrounding areas. The effect of air pollution on COVID-19 spread and severity is likely different in different regions. In sum, so that the overall estimate of the effect is not influenced heavily by outliers and smaller areal unit estimates are pulled toward the mean, we recommend a random slope model or meta-analysis with random effects for future articles.

#### Modeling.

A large fraction (~25%) of the papers reviewed did not control for other factors. Cross-sectional studies will have a substantial amount of bias if they do not control for covariates. Twenty-one of the 78 cross-sectional papers did not control for any other factors in their models. These papers conducted either a correlation analysis or a univariate linear regression, and readers should understand and interpret results within this context. Nineteen of the 61 short-term time series papers did not include covariates. Time series studies will also introduce some degree of bias and will also have higher variance in their estimates by choosing not to control for other covariates because air pollution levels are correlated with meteorological variables such as temperature, wind speed/direction, humidity, and rainfall (48).

Of the 64 cross-sectional studies that employed regression approaches, the majority of them used linear regression followed by negative binomial regression and Poisson regression. The normality assumption from linear regression generally holds when each individual unit has a large population. However, especially when using United States counties, LSOAs in England (34), or municipalities in Italy (14), the normality assumption may not hold because the number of people in each unit can be fewer than 100 and the COVID-19 cases and deaths could be zero or close to zero. In these settings, methods for modeling count data such as negative binomial regression or Poisson regression may be necessary (33 papers used these models). Another key consideration is whether the relationship between air pollution and COVID-19 cases and deaths is linear in nature. It is likely that the relationship is nonlinear, which means negative binomial and Poisson models may be more appropriate. In addition, 11 of the papers employed generalized additive models (GAM) to capture this nonlinear relationship. Chakrabarty et al. (11), for example, explore the relationship between air pollution and COVID-19 using GAMs on their cross-sectional data. Wang et al. (60) used GAMs for their time series study. GAMs are quite flexible, but they do not generally provide a numerical result for the relationship between air pollution and COVID-19. The primary result is instead a visualization.

Finally, the association of air pollution with COVID-19 is likely not constant across geographical areas within a country, and it is likely that areas close to each other have similar effects. One can account for these geographic differences in the relationship in many ways. Four of the 139 papers used geographically weighted regression (32, 43, 46, 66) to account for geographical variations. In this approach, parameters vary continuously across the entire area of study. A similar approach would be a hierarchical model with a random slope component in which the relationship between air pollution and COVID-19 varies at a geographic unit of area that is larger (e.g., states) than the smallest unit of area considered in the study (e.g., counties).

#### Reproducibility.

To discover crucial linkages between air pollution and adverse COVID-19 outcomes in a more definitive, causal manner, both the data used for the analyses as well as the code should be made publicly available. Transparency and shared resources will assist in the global push toward uncovering solutions to the COVID-19 pandemic and instituting public policies that will protect the health of people worldwide. Only 8 of the 139 selected papers in our evidence synthesis included publicly available code. Because this research area is rapidly developing, access to code will be critical to validate results and build on the conclusions. It is difficult to reproduce results and continue work on these areas without code availability. In Wu et al. (61), we made both the data and code publicly available.

### Geographical Variation and the Need for Data Visualization

A majority (83 papers) of the 139 papers reviewed studied the relationship between air pollution and COVID-19 in Italy, the United States, and China. Most of the remaining papers studied air pollution and COVID-19 in Western Europe and South Asia. Based on the existing studies, the relationship between air pollution and COVID-19 likely varies around the world. As a result, findings from studies conducted primarily in three countries are not necessarily generalizable to other countries, particularly those with different climates. It is particularly noticeable that none of the 139 papers analyzed data from Africa, Russia, Australia, a majority of Eastern European countries, Middle Eastern countries, and South American countries. These gaps in the literature were easily identified despite the large number of studies because of the dashboard we created, which was able to effectively summarize and visualize the study designs and results.

### Importance of the Visualization and Evidence Synthesis Pipeline

Our proposed approach to the visualization of evidence synthesis uses an innovative, interactive dashboard that allows the user to learn about the characteristics of any single study included in the evidence base as well as to synthesize the evidence in various important ways. First, it enables an interactive geographical visualization of where the evidence is coming from, which allows us to easily identify underrepresented populations. Second, it allows a temporal visualization of how the evidence is accumulating in the context of a rapidly evolving area of research such as the COVID-19 pandemic. Third, our approach enables the research community to easily digest complex information from highly heterogeneous studies of different pollutants, different outcomes, and different study designs. For example, users can choose to visualize studies that meet specific criteria or that focus on a single pollutant (e.g., PM_2.5_). Most importantly, it enables other investigators to manually upload peer-reviewed studies, allowing the evidence synthesis to update dynamically as new studies on the topic are published. Finally, this approach is transferable to other contexts, and it can be used broadly to summarize evidence from heterogeneous studies in any area of research.

This study demonstrates that geography in evidence synthesis matters: Air pollution exposures and how those exposures interact with the health burden in the community differ based on where an individual is located. Therefore, our visualization approach can more clearly represent the nature of the research associated with a particular region, making it easier to visualize where there is a dearth of research and where additional research is needed to paint a fuller picture of the relationship between air pollution and COVID-19 cases and deaths. Visualizing a body of research is essential for understanding the spatial distribution and patterns in the evidence that will inform future research efforts. This community-focused evidence synthesis approach has formative effects on how we move forward and publish papers as an international research community. Furthermore, from a perspective of equity and representativeness, where studies have been conducted becomes a more pronounced aspect of the work, and metadata on the geographic area of study could become a requirement of publishing papers; automation would make this process significantly easier.

## CONCLUSIONS

Assessing the short- and long-term effects of air pollution on adverse COVID-19 health outcomes is a rapidly evolving area of research, and many more studies will likely be published in the coming months and years. For existing studies, we have reviewed the evidence and identified the statistical challenges that researchers have faced when analyzing the relationship between air pollution and COVID-19, and we have also offered recommendations for future studies. To account for the fact that there will be hundreds of additional papers published on this topic in the future, we also describe a new approach to synthesizing the accumulated evidence in an interactive and dynamic manner. Our innovative approach to evidence synthesis allows for the visualization of spatial and temporal patterns in research that can be applied across many research areas.

## Supplementary Material

Supplemental Material

supplemental material_a

supplemental material_b

## Figures and Tables

**Table 1:** Types of information products produced by the automated evidence synthesis

Type	Purpose	Question
Location map	Shows the spatial extent and concentration of the research	Do research study areas include all impacted populations or varieties of the coronavirus?
Temporal animation	Shows patterns of publication across space and time	Does research represent all waves of COVID-19?
Map with filters	Allows the user to filter based on pollutant, number of citations, or statistical method	Has a statistical method been used broadly?
Standalone Web application	Is intended for general users (nonresearchers). Shows the same information as other products but in a more user-friendly way	Are the scale and scope of the research understandable to the general public?

## Data Availability

All data needed to evaluate the conclusions in the article are present in the article and/or the Supplemental Materials. Data are available in Supplemental Table 2. Code is available at http://bit.ly/3UOY2SY.
