# Supplementary material for: A Literature Review of the Effects of Air Pollution on COVID-19 Health Outcomes Worldwide: Statistical Challenges and Data Visualization
Source: Annu Rev Public Health. Author manuscript; Available in PMC 2024 Nov 15. (PMC11567163; doi:10.1146/annurev-publhealth-071521-120424)
Supplement: Supplemental Material [file NIHMS2031411-supplement-Supplemental_Material.pdf]

A New Approach for Evidence Synthesis

Import and potentially install necessary Python packages.

In [1]:

```
import sys
import pandas as pd
import json
import time
import datetime

try:
    import crossref_commons.retrieval
except ImportError as e:
    print('Required module crossref_commons not found: reinstalling')
    t = time.process_time()
    !{sys.executable} -m pip install crossref_commons
    elapsed_time = time.process_time() - t
    import crossref_commons.retrieval
    print('Installation time (hh:mm:ss): ' + str(datetime.timedelta(seconds=elapsed_t

try:
    from habanero import cn
    #from habanero import counts
except ImportError as e:
    print('Required module habanero not found: reinstalling')
    t = time.process_time()
    !{sys.executable} -m pip install habanero
    elapsed_time = time.process_time() - t
    from habanero import cn
    #from habanero import counts
    print('Installation time (hh:mm:ss): ' + str(datetime.timedelta(seconds=elapsed_t

try:
    from semanticscholar import SemanticScholar
except ImportError as e:
    print(print('Required module SemanticScholar not found: reinstalling'))
    t = time.process_time()
    !{sys.executable} -m pip install semanticscholar
    elapsed_time = time.process_time() - t
    from semanticscholar import SemanticScholar
    print('Installation time (hh:mm:ss): ' + str(datetime.timedelta(seconds=elapsed_t

try:
    from crossref.restful import Works
except ImportError as e:
    print('Required module habanero not found: reinstalling')
    t = time.process_time()
    !{sys.executable} -m pip install crossrefapi
    elapsed_time = time.process_time() - t
    from crossref.restful import Works
    print('Installation time (hh:mm:ss): ' + str(datetime.timedelta(seconds=elapsed_t
```

Read the literature review spreadsheet and create a data frame.

In [2]:

```
dataset = '/arcgis/home/all_papers_new_format_version_5_kab.xlsx'
```

```
[illegible]
df = pd.read_excel(dataset)
df = df[df['DOI'].notna()]
uniqueID = df.groupby(['DOI']).ngroup()
df.insert(0, "ID", uniqueID)
df.head()
```

In [4]:

```
def getAuthors(namesDic):
    #print(namesDic)
    numAuthors = len(namesDic)

    namesList = ""
    for i in range(numAuthors):
        try:
            first = namesDic[i]["given"]
        except KeyError as e:
            first = ''
        try:
            last = namesDic[i]['family']
        except KeyError as e:
            last = ''
        namesList += first + " " + last
        if i != numAuthors - 1:
            namesList += ", "

    return namesList
```

For each unique DOI, find the author information (affiliation) and generate an APA formatted citation.

In [10]:

```
uniqueDOIs = df["DOI"]

title = []
authors = []
citation = []
year = []
publishDates = []
citationCountsHabanero = []

for doi in uniqueDOIs:
    print("Checking DOI: " + doi)
    # Get JSON of the paper from CrossRef
    paperJSON = crossref_commons.retrieval.get_publication_as_json(doi)

    # Get papers title
    title.append(paperJSON['title'][0])

    # Get Authors names
    # print(paperJSON['author'])
    names = getAuthors(paperJSON['author'])
    authors.append(names)

    # Get Citations
    apaCitation = cn.content_negotiation(doi, format="text", style="apa")
    citation.append(apaCitation)

    # Get year published
    [illegible]
```

```
    year.append(paperJSON['created']['date-parts'][0][0])
    YYYY = paperJSON['created']['date-parts'][0][0]
    MM = paperJSON['created']['date-parts'][0][1]
    DD = paperJSON['created']['date-parts'][0][2]
    publishDates.append(datetime.datetime(YYYY, MM, DD))

    # Get the citation counts from habanero package
    # print(counts.citation_count(doi))
    try:
        citationCountsHabanero.append(paperJSON['is-referenced-by-count'])
    except:
        citationCountsHabanero.append(None)
        pass
```

In [11]:

```
df['Paper Title'] = title
df['Authors'] = authors
df['Year'] = year
df['Citation (APA)'] = citation
df['Citation Counts Habanero'] = citationCountsHabanero
df.head()
#output.to_csv("output.csv", index=False)
```

Out[11]:

	ID	DOI	Study Area Country	Study Area Admin Level 1	Paper Title	Authors	Year	Citation (APA)	Ha
29	0	https://doi.org/10.1007/s10640-020-00486-1	Italy	NaN	The Effects of Air Pollution on COVID-19 Relat...	Eric S. Coker, Laura Cavalli, Enrico Fabrizi, ...	2020	Coker, E. S., Cavalli, L., Fabrizi, E., Guaste...	
15	1	https://doi.org/10.1007/s10640-020-00491-4	Netherlands	Aa en Hunze, Aalsmeer, Aalten, Achtkarspelen, ...	Air Pollution Exposure and Covid-19 in Dutch M...	Matthew A. Cole, Ceren Ozgen, Eric Strobl	2020	Cole, M. A., Ozgen, C., & Strobl, E. (2020). A...	
13	2	https://doi.org/10.1007/s10668-020-00878-9	India, China, Pakistan, Indonesia	NaN	Air pollution aggravating COVID-19 lethality? ...	Ankit Gupta, Hemant Bherwani, Sneha Gautam, Sa...	2020	Gupta, A., Bherwani, H., Gautam, S., Anjum, S....	
53	3	https://doi.org/10.1007/s40201-020-00564-y	China	NaN	Correlations between Meteorological Indicators...	Huiying Huang, Xiuji Liang, Jingxiu Huang, Zha...	2020	Huang, H., Liang, X., Huang, J., Yuan, Z., Ouy...	
		https://			Ambient nitrogen	Ye Yao, Jinhua		Yao, Y., Pan, J.,	

**57**	4	doi.org/10.1016/ j.ecoenv.2020.111421	China	NaN	dioxide pollution and spreada...	Pan, Zhixi Liu, Xia Meng, Weido...	2020	Liu, Z., Meng, X., Wang, W.,...

Split each paper based on their country and their corresponding admin level1

In [13]:

```
def tidy_split(df, column, sep='|', keep=False):
    """
    Split the values of a column and expand so the new DataFrame has one split
    value per row. Filters rows where the column is missing.

    Params
    ------
    df : pandas.DataFrame
        dataframe with the column to split and expand
    column : str
        the column to split and expand
    sep : str
        the string used to split the column's values
    keep : bool
        whether to retain the presplit value as it's own row

    Returns
    -------
    pandas.DataFrame
        Returns a dataframe with the same columns as `df`.
    """
    indexes = list()
    new_values = list()
    df = df.dropna(subset=[column])
    for i, presplit in enumerate(df[column].astype(str)):
        values = presplit.split(sep)
        if keep and len(values) > 1:
            indexes.append(i)
            new_values.append(presplit)
        for value in values:
```
