# Supplementary material for: A Literature Review of the Effects of Air Pollution on COVID-19 Health Outcomes Worldwide: Statistical Challenges and Data Visualization
Source: Annu Rev Public Health. Author manuscript; Available in PMC 2024 Nov 15. (PMC11567163; doi:10.1146/annurev-publhealth-071521-120424)
Supplement: supplemental material_a [file NIHMS2031411-supplement-supplemental_material_a.pdf]

Paper #	Title	Citation APA	DOI	Author	Publication Year	Publication date	Temporal Extent	Geographical Area of Study	Areal unit	Cases	Deaths	Reproductive ratio	Case fatality rate	Hospitalizations	Other outcome	Statistical methods	PM2.5	PM10	O3	NO2	SO2	CO	AQI	Other air pollutant	Covariates or Possible Confounders	Code Availability (link)
1	Assessing nitrogen dioxide (NO_2) levels as a contributing factor to the coronavirus (COVID-19) fatality rate (*Science of the Total Environment*)	Ogen, Y. (2020). Assessing nitrogen dioxide (NO2) levels as a contributing factor to coronavirus (COVID-19) fatality. *Science of the Total Environment* , *726* , 138605.	https://doi.org/10.1016/j.scitotenv.2020.138605	Ogen	2020	4/11/2020	1	Italy, Spain, France, G	66 Administrative Region		1					Descriptive	0	0	0	2	0	0	0		None	No
2	Incidence of COVID-19 and connections with air pollution exposure: evidence from the Netherlands (medRxiv)	Andrée, B. P. J. (2020). Incidence of COVID-19 and connections with air pollution exposure: evidence from the Netherlands. *World Bank Policy Research Working Paper* , (9221).	https://doi.org/10.1596/1813-9450-9448	Andree	2020	12/3/2020	1	Netherlands	355 municipalities	1				1		Non-parametric penalized kernel regression	2	0	0	0	0	0	0		Population density, gender, age structure, pro	No
3	Initial evidence of higher morbidity and mortality due to SARS-CoV-2 in regions with lower air quality (Frontiers in Public Health)	Pansini, R., & Fornacca, D. (2020). Initial evidence of higher morbidity and mortality due to SARS-CoV-2 in regions with lower air quality. *Frontiers of Public Health* .	10.3389/fpubh.2020.597753	Pansini and Fornacca	2021	1/22/2021	1	United States, Italy, C	United States: counties, It	1	1					Kendall tau correlation	2	2	2	0	1	2	0	HCHO- 2, Aerosol- 1	None	No
4	Early Spread of COVID-19 in the Air-Polluted Regions of Eight Severely Affected Countries (Atmosphere)	Pansini, R., & Fornacca, D. (2021). Early spread of COVID-19 in the air-polluted regions of eight severely affected countries. *Atmosphere* , *12* (6), 795.	https://doi.org/10.3390/atmos12060795	Pansini and Fornacca	2021	6/21/2021	1	China, Italy, Iran, Unit	equivalent of county-level	1	1		1			Kendall tau correlation	2	2	1	2	1	2	0		None	No
5	Urban air pollution may enhance COVID-19 case fatality and mortality rates in the United States (The Innovation)	Liang, D., Shi, L., Zhao, J., Liu, P., Sarnat, J. A., Gao, S., ... & Chang, H. H. (2020). Urban air pollution may enhance COVID-19 case-fatality and mortality rates in the United States. *The Innovation* , *1* (3), 100047.	https://doi.org/10.1016/j.xinn.2020.100047	Liang et al.,	2020	9/21/2020	1	United States	3122 counties				1			Negative binomial mixed model	1	0	1	2	0	0	0		State-level test positive rate, county-level hea	No
6	Air Pollution and COVID-19 Mortality in the United States: Strengths and Limitations of an Ecological Regression Analysis (Science Advances)	Wu, X., Nethery, R. C., Sabath, M. B., Braun, D., & Dominici, F. (2020). Air pollution and COVID-19 mortality in the United States: Strengths and limitations of an ecological regression analysis. *Science advances* , *6* (45), eabd4049.	https://doi.org/10.1126/sciadv.abd4049	Wu et al.,	2020	11/5/2020	1	United States	3089 counties		1					Negative binomial mixed model	2	0	0	0	0	0	0		Days since first COVID-19 case reported (epid	Yes (https://github.com/wxwx1993/PM_COVID)
7	Links between air pollution and COVID-19 in England (Environmental Pollution)	Travaglio, M., Yu, Y., Popovic, R., Selley, L., Leal, N. S., & Martins, L. M. (2021). Links between air pollution and COVID-19 in England. *Environmental Pollution* , *268* , 115859.	https://doi.org/10.1016/j.envpol.2020.115859	Travaglio et al.,	2020	10/19/2020	1	England	7 regions, 343 local-autho	1	1					Multivariate negative binomial regression	2	2	1	2	2	0	0	NO -2	Regional and sub regional level: population d	Yes (https://github.com/M1gus/AirPollutionCOVID19, https://m1gus.github.io/AirPollutionCOVID19/)
8	Risk of COVID-19 is associated with long-term exposure to air pollution (medRxiv)	Zheng, P., Liu, Y., Song, H., Wu, C. H., Li, B., Kraemer, M. U., ... & Jia, G. (2020). Risk of COVID-19 is associated with long-term exposure to air pollution.	https://doi.org/10.1101/2020.04.21.20073700	Zheng et al.,	2020	4/25/2020	1	China	324 cities	1					1	Multivariate Poisson regression model	2	1	1	2	1	1	0		Gross domestic product per capita, age comp	No
9	Role of the chronic air pollutions in the COVID-19 outbreak risk in Italy (Environmental Pollution)	Fattorini, D., & Regoli, F. (2020). Role of the chronic air pollution levels in the Covid-19 outbreak risk in Italy. *Environmental pollution* , *264* , 114732.	https://doi.org/10.1016/j.envpol.2020.114732	Fattorini and Regoli	2020	5/4/2020	1	Italy	71 provinces	1						Univariate linear regression	2	2	2	2	0	0	0		None	No
10	Factors determining the diffusion of COVID-19 and suggested strategy to prevent future accelerated viral infectivity similar to COVID (Sci Total Env)	Coccia, M. (2020). Factors determining the diffusion of COVID-19 and suggested strategy to prevent future accelerated viral infectivity similar to COVID. *Science of the Total Environment* , *729* , 138474.	https://doi.org/10.1016/j.scitotenv.2020.138474	Coccia et al.,	2020	4/20/2020	1	Italy	55 provincial capitals	1						Multiple linear regression	0	2	2	0	0	0	0		Temperature, percent moisture, wind speed	No
11	Association between environmental pollution and prevalence of coronavirus disease 2019 (COVID-19) in Italy (medRxiv)	Lippi, G., Sanchis-Gomar, F., & Henry, B. M. (2020). Association between environmental pollution and prevalence of coronavirus disease 2019 (COVID-19) in Italy. *MedRxiv* .	https://doi.org/10.1101/2020.04.22.20075986	Lippi et al.,	2020	4/27/2020	1	Italy	provinces with greater tha	1						Univariate linear regression	0	2	2	0	0	0	0		None	No
12	Risk factors associated with mortality of COVID-19 in 3125 counties of the United States (Infectious Disease of Poverty)	Tian, T., Zhang, J., Hu, L., Jiang, Y., Duan, C., Li, Z., ... & Zhang, H. (2021). Risk factors associated with mortality of COVID-19 in 3125 counties of the United States. *Infectious diseases of poverty* , *10* (1), 1-8.	https://doi.org/10.1186/s40249-020-00786-0	Tian et al.,	2021	1/4/2021	1	United States	2692 counties		1					Partitioning Around Medoids (PAM) clustering algorithm and multivariate negative binomial regression	2	0	0	0	0	0	0		Proportion with access to exercise opportunit	No
13	Air Pollution aggravating COVID-19 lethality? Exploration in Asian Cities using Statistical Models (Environment, Development, and Sustainability)	Gupta, A., Bherwani, H., Gautam, S., Anjum, S., Musugu, K., Kumar, N., ... & Kumar, R. (2021). Air pollution aggravating COVID-19 lethality? Exploration in Asian cities using statistical models. *Environment, Development and Sustainability* , *23* (4), 6408-6417.	https://doi.org/10.1007/s10668-020-00878-9	Gupta et al,	2020	7/15/2020	1	India, China, Pakistan,	3 Indian Cities, 3 Chinese	1	1					Univariate Linear Regression	2	1	0	0	0	0	0		None	No
14	Air Pollution exposure and COVID-19: A look at mortality in Mexico City using individual-level data (Science of the Total Environment)	López-Feldman, A., Heres, D., & Marquez-Padilla, F. (2021). Air pollution exposure and COVID-19: a look at mortality in Mexico City using individual-level data. *Science of the Total Environment* , *756* , 143929.	https://doi.org/10.1016/j.scitotenv.2020.143929	Lopez-Feldman et al,	2020	11/26/2020	1	Mexico City	196,273 individuals in the Mexico City Metropoli		1					Probit Model	2	0	0	0	0	0	0		Municipal-Level: population density, density	No
15	Air Pollution Exposure and COVID-19 in Dutch Municipalities (Environmental and Resource Economics)	Cole, M. A., Ozgen, C., & Strobl, E. (2020). Air pollution exposure and Covid-19 in Dutch municipalities. *Environmental and Resource Economics* , *76* (4), 581-610.	https://doi.org/10.1007/s10640-020-00491-4	Cole et al,	2020	8/4/2020	1	Netherlands	355 municipalities	1	1			1		Negative Binomial	2	0	0	1	1	0	0		Proximity to carnival events, bordering Germa	No
16	Air Pollution is Associated with COVID-19 Incidence and Mortality in Vienna Austria (International Journal of Environmental Research and Public Health)	Hutter, H. P., Poteser, M., Moshammer, H., Lemmerer, K., Mayer, M., Weitensfelder, L., ... & Kundi, M. (2020). Air pollution is associated with COVID-19 incidence and mortality in Vienna, Austria. *International Journal of Environmental Research and Public Health* , *17* (24), 9275.	https://doi.org/10.3390/ijerph17249275	Hutter et al,	2020	12/14/2020	1	Vienna	Austria: 23 Districts	1	1					Cox Proportional Hazard	0	2	0	2	0	0	0		Percent aged 65+, percent foreigners, unemp	No
17	Air Pollution, sociodemographic and health conditions effects on COVID-19 mortality in Colombia: An ecological study (Science of the Total Environment)	Rodriguez-Villamizar, L. A., Belalcazar-Ceron, L. C., Fernández Niño, J. A., Marín-Pineda, D. M., Rojas-Sánchez, O. A., Acuña-Merchán, L. A., ... & Herrera-Galindo, V. M. (2021). Air pollution, sociodemographic and health conditions effects on COVID-19 mortality in Colombia: an ecological study. *Science of the Total Environment* , *756* , 144020.	https://doi.org/10.1016/j.scitotenv.2020.144020	Rodriguez-Villamizar et al,	2020	11/26/2020	1	Columbia	1122 municipalities		1				1	Logit-Negative Binomial Hurdle Model	1	0	0	0	0	0	0		Percent aged 65+, percent urban population,	No
18	Ambient Air Pollutants and their Effects on COVID-19 Mortality in the United States of America	Liu, S., & Li, M. (2020). Ambient air pollutants and their effect on COVID-19 mortality in the United States of America. *Revista Panamericana de Salud Pública* , *44* .	https://doi.org/10.26633/RPSP.2020.159	Liu and Li,	2020	11/20/2020	1	United States	2522 counties		1					Multivariate Linear Regression	0	0	2	2	2	1	0		Median household income, number of hospit	No
19	A Model of Disparities: Risk Factors Associated with COVID-19 Infection	Rozenfeld, Y., Beam, J., Maier, H., Haggerson, W., Boudreau, K., Carlson, J., & Medows, R. (2020). A model of disparities: risk factors associated with COVID-19 infection. *International journal for equity in health* , *19* (1), 1-10.	https://doi.org/10.1186/s12939-020-01242-z	Rozenfeld et al,	2020	7/29/2020	1	United States	34,503 cases of laboratory confirmed positive or negative COVID-19 infections						1	Multivariate Logistic Regression	0	0	0	0	0	0	2		Age, gender, education, employment, race, et	No
20	Association Between Air Pollution in Lima and the High Incidence of COVID-19: Findings from a Post Hoc Analysis (BMC Public Health)	Vasquez-Apestegui, B. V., Parras-Garrido, E., Tapia, V., Paz-Aparicio, V. M., Rojas, J. P., Sanchez-Ccoyllo, O. R., & Gonzales, G. F. (2021). Association between air pollution in Lima and the high incidence of COVID-19: findings from a post hoc analysis. *BMC Public Health* , *21* (1), 1-13.	https://doi.org/10.1186/s12889-021-11232-7	Vasquez-Apestegui et al,	2021	6/16/2021	1	Peru	24 districts in Lima	1	1		1			Linear Regression	2	0	0	0	0	0	0		Sex ratio, age, food markets, population, elev	No
21	Geographical Pattern of COVID-19-Related Outcomes over the Pandemic Period in France: A Nationwide Socio-Environmental Study	Deguen, S., & Kihal-Talantikite, W. (2021). Geographical pattern of COVID-19-Related outcomes over the pandemic period in France: a nationwide Socio-Environmental study. *International Journal of Environmental Research and Public Health* , *18* (4), 1824.	https://doi.org/10.3390/ijerph18041824	Séverine Deguen and Wahida Kihal-Talantikite,	2021	2/14/2021	1	France	95 Departments		1			1	1	Linear Regression	0	0	0	2	0	0	0		Proportion of population over 60 and populat	No
22	Are environmental pollution and biodiversity levels associated to the spread and mortality of COVID-19? A four-month global analysis	Fernández, D., Giné-Vázquez, I., Liu, I., Yucel, R., Ruscone, M. N., Morena, M., ... & Tyrovolas, S. (2021). Are environmental pollution and biodiversity levels associated to the spread and mortality of COVID-19? A four-month global analysis. *Environmental pollution* , *271* , 116326.	https://doi.org/10.1016/j.envpol.2020.116326	D. Fernandez, I.Gine-Vazquez, I. Liu et al.,	2020	12/21/2020	1	218 countries		1	1					Spatio temporal models	2	2	2	0	0	0	0		Days since the first case, Days since the last ca	No
23	Vulnerability and Burden of All-Cause Mortality Associated with Particulate Air Pollution during COVID-19 Pandemic: A Nationwide Observed Study in Italy	Ye, T., Xu, R., Yu, W., Chen, Z., Guo, Y., & Li, S. (2021). Vulnerability and burden of all-cause mortality associated with particulate air pollution during covid-19 pandemic: a nationwide observed study in Italy. *Toxics* , *9* (3), 56.	https://doi.org/10.3390/toxics9030056	Tingting Ye,Rongbin Xu, Wenhua Yu, Zhaoyue Chen, Yuming Guo, and Shanshan Li	2021	3/16/2021	1	Italy	107 provinces	1	1					A time stratified case-cross design was utilized alongside a distributed lag nonlinear model	2	2	0	0	0	0	0		Temperature, Relative humidity, NO2, CO, O3	No
24	Population-weighted exposure to air pollution and COVID-19 incidence in Germany	Huang, G., & Brown, P. E. (2021). Population-weighted exposure to air pollution and COVID-19 incidence in Germany. *Spatial statistics* , *41* , 100480.	https://doi.org/10.1016/j.spasta.2020.100480	G Huang, PE Brown	2020	11/3/2020	1	Germany	401 administrative Distric	1						Poisson Log Linear Model	1	1	0	2	1	0	0		Population Density, Temperature, Age, Sex	Yes (https://github.com/hgw0610209/Germany-covid-paper)
25	Long-term exposure to air-pollution and COVID-19 mortality in England: A hierarchical spatial analysis	Konstantinoudis, G., Padellini, T., Bennett, J., Davies, B., Ezzati, M., & Blangiardo, M. (2021). Long-term exposure to air-pollution and COVID-19 mortality in England: a hierarchical spatial analysis. *Environment international* , *146* , 106316.	https://doi.org/10.1016/j.envint.2020.106316	G Konstantinoudis, T Padellini, J Bennett	2020	12/7/2020	1	England	32,844 LSOAs		1					Bayesian Hierarchical Poisson Log-Linear Model	1	0	0	1	0	0	0		Many relevant confounders including meteor	Yes (https://github.com/gkonstantinoudis/COVID19AirpollutionEn)
26	Effects of air pollution on the potential transmission and mortality of COVID-19: A preliminary case-study in Tarragona Province (Catalonia, Spain)	Marquès, M., Rovira, J., Nadal, M., & Domingo, J. L. (2021). Effects of air pollution on the potential transmission and mortality of COVID-19: a preliminary case-study in Tarragona Province (Catalonia, Spain). *Environmental Research* , *192* , 110315.	https://doi.org/10.1016/j.envres.2020.110315	M Marquès, J Rovira, M Nadal, JL Domingo	2020	10/12/2020	1	Spain	Taragona Province	1	1					Pearson Correlation	1	0	1	2	0	0	0		None	No
27	Regional and global contributions of air pollution to risk of death from COVID-19	Pozzer, A., Dominici, F., Haines, A., Witt, C., Münzel, T., & Lelieveld, J. (2020). Regional and global contributions of air pollution to risk of death from COVID-19. *Cardiovascular Research* , *116* (14), 2247-2253.	https://doi.org/10.1093/cvr/cvaa288	A Pozzer, F Dominici, A Haines, C Witt	2020	10/1/2020	1	East Asia, Europe, North America, Africa, West Asia, South Asia, South			1					Exposure response function of the WHO to estimate relative risk	2	0	0	0	0	0	0		None	No
28	The Effects of Air Pollution on COVID-19 Related Mortality in Northern Italy	Coker, E. S., Cavalli, L., Fabrizi, E., Guastella, G., Lippo, E., Parisi, M. L., ... & Vergalli, S. (2020). The effects of air pollution on COVID-19 related mortality in northern Italy. *Environmental and Resource Economics* , *76* (4), 611-634.	https://doi.org/10.1007/s10640-020-00486-1	ES Coker, L Cavalli, E Fabrizi, G Guastella	2020	8/4/2020	1	Italy	Northern Italy: 4041 municipalities						1	Negative Binomial Regression	2	0	0	0	0	0	0		Population Density, Income, Percent Industrial Land, Percent of Companies with less than 10 employees, Temperature, Male Percentage, Age, Hospital Beds, Number of university students, Share of EU residents	
29	Air pollutants and risk of death due to COVID-19 in Italy (Environmental Research)	Dettori, M., Deiana, G., Balletto, G., Borruso, G., Murgante, B., Arghittu, A., ... & Castiglia, P. (2021). Air pollutants and risk of death due to COVID-19 in Italy. *Environmental Research* , *192* , 110459.	https://doi.org/10.1016/j.envres.2020.110459	M Dettori, G Deiana, G Balletto, G Borruso	2020	11/11/2020	1	Italy	107 provinces						1	Linear Regression	1	2	0	1	0	0	0		Tree Density, Pedestrianized Areas, Soil Consu	No
30	Initial COVID-19 Transmissibility and Three Gaseous Air Pollutants (NO2, SO2, and CO): a Nationwide Ecological Study in China	Ran, J., Zhao, S., Han, L., Peng, Z., Wang, M. H., Qiu, Y., & He, D. (2020). Initial COVID-19 transmissibility and three gaseous air pollutants (NO2, SO2, and CO): a nationwide ecological study in China. *Frontiers in medicine* , 571.	https://doi.org/10.3389/fmed.2020.575839	J Ran, S Zhao, L Han, Z Peng, MH Wang, Y Qiu	2020	9/24/2020	1	China	154 cities			1				Linear Regression	0	0	0	1	1	1	0		Temperature and Humidity	No
31	Hazardous air pollutant exposure as a contributing factor to COVID-19 mortality in the United States (Environmental Research Letters)	Petroni, M., Hill, D., Younes, L., Barkman, L., Howard, S., Howell, I. B., ... & Collins, M. B. (2020). Hazardous air pollutant exposure as a contributing factor to COVID-19 mortality in the United States. *Environmental Research Letters* , *15* (9), 0940a9.	https://doi.org/10.1088/1748-9326/abaf86	M Petroni, D Hill, L Younes, L Barkman	2020	9/11/2020	1	United States	3006 counties		1					Negative Binomial Mixed Models	2	0	2	0	0	0	0	Hazardous Air Pollutants- 2, Acetaldehyde- 2, Naphthalene		No
32	Particulate matter pollution and the COVID-19 outbreak: results from Italian regions and provinces (Archives of Medical Science)	Bianconi, V., Bronzo, P., Banach, M., Sahebkar, A., Mannarino, M. R., & Pirro, M. (2020). Particulate matter pollution and the COVID-19 outbreak: results from Italian regions and provinces. *Archives of Medical Science: AMS* , *16* (5), 985.	https://doi.org/10.5114/aoms.2020.95336	V Bianconi, P Bronzo, M Banach	2020	5/22/2020	1	Italy	20 regions	1	1					Multiple Linear Regression	2	2	0	0	0	0	0		Age, Sex, Temperature, Humidity	No
33	COVID-19 prevalence and fatality rates in association with air pollution emission concentrations and emission sources (Environmental Pollution)	Hendryx, M., & Luo, J. (2020). COVID-19 prevalence and fatality rates in association with air pollution emission concentrations and emission sources. *Environmental Pollution* , *265* , 115126.	https://doi.org/10.1016/j.envpol.2020.115126	M Hendryx, J Luo	2020	6/27/2020	1	United States	3143 counties	1	1					Mixed Model	2	0	1	0	0	0	0	Diesel Particulate Matter- 2	Age, Race, Ethnicity, Education, Income, Smok	No
34	Ambient fine particulate matter air pollution and the risk of hospitalization among COVID-19 positive individuals: Cohort study (Environment International)	Bowe, B., Xie, Y., Gibson, A. K., Cai, M., van Donkelaar, A., Martin, R. V., ... & Al-Aly, Z. (2021). Ambient fine particulate matter air pollution and the risk of hospitalization among COVID-19 positive individuals: cohort study. Environment international, 154, 106564.	https://doi.org/10.1016/j.envint.2021.106564	B Bowe, Y Xie, AK Gibson, M Cai	2021	4/9/2021	1	United States	169,102 United States Veterans					1		Poisson Regression	2	0	0	0	0	0	0		State; Age, Race, Sex, Smoking Status, County	No
35	The IMPACT of LONG-TERM Exposure to PM2.5, PM10 and NO2 Air Pollutants on the Age-Adjusted Mortality RATE of COVID-19 Based on the Example of Poland (Value in Health)	Musiałek, A., & Nosowicz, A. (2020). PIN120 The IMPACT of LONG-TERM Exposure to PM2. 5, PM10 and NO2 Air Pollutants on the Age-Adjusted Mortality RATE of COVID-19 Based on the Example of Poland. *Value in Health* , *23* , S563-S564.	https://doi.org/10.1016/j.jval.2020.08.961	A Musialek, A Nosowicz	2020	12/11/2020	1	Poland	16 Voivodeships		1					Linear Regression	1	1	0	2	0	0	0		None	No
36	The main factors influencing COVID-19 spread and deaths in Mexico: A comparison between phases I and II	Benita, F., & Gasca-Sanchez, F. (2021). The main factors influencing COVID-19 spread and deaths in Mexico: A comparison between phases I and II. *Applied Geography* , *134* , 102523.	https://doi.org/10.1016/j.apgeog.2021.102523	F Benita, F Gasca-Sanchez	2021	7/27/2021	1	Mexico	2459 municipalities in 32	1	1					Zero-Inflated Negative Binomial Regression Models	2	0	0	0	0	0	0		None	No
37	Effects of long-term exposure to air pollutants on the spatial spread of COVID-19 in Catalonia, Spain (Environmental Research)	Saez, M., Tobias, A., & Barceló, M. A. (2020). Effects of long-term exposure to air pollutants on the spatial spread of COVID-19 in Catalonia, Spain. *Environmental research* , *191* , 110177.	https://doi.org/10.1016/j.envres.2020.110177	M Saez, A Tobias, MA Barceló	2020	9/12/2020	1	Spain	Catalonia	1	1					Poison Mixed Models	0	2	0	2	0	0	0		Income, Unemployment, Age, Percent Foreign	No
38	An ecological analysis of long-term exposure to PM2.5 and incidence of COVID-19 in Canadian Health Regions (Environmental Research)	Stieb, D. M., Evans, G. J., To, T. M., Brook, J. R., & Burnett, R. T. (2020). An ecological analysis of long-term exposure to PM2. 5 and incidence of COVID-19 in Canadian health regions. *Environmental research* , *191* , 110052.	https://doi.org/10.1016/j.envres.2020.110052	DM Stieb, GJ Evans, TM To, JR Brook	2020	8/26/2020	1	Canada	111 health regions	1						Negative Binomial Regression	2	0	0	0	0	0	0		Population density, days since first case, days	No
39	County-level Predictors of Coronavirus Disease 2019 (COVID-19) Cases and Deaths in the United States: What Happened, and Where Do We Go from Here? (Clinical Infectious Diseases)	McLaughlin, J. M., Khan, F., Pugh, S., Angulo, F. J., Schmitt, H. J., Isturiz, R. E., ... & Swerdlow, D. L. (2021). County-level Predictors of Coronavirus Disease 2019 (COVID-19) Cases and Deaths in the United States: What Happened, and Where Do We Go from Here?. *Clinical Infectious Diseases* , *73* (7), e1814-e1821.	https://doi.org/10.1093/cid/ciaa1729	JM McLaughlin, F Khan, S Pugh	2020	11/16/2020	1	United States	3142 counties	1	1					Negative Binomial Mixed Model	2	2	0	0	0	0	0		Age, Race, Income, Unemployment, Comorbid	No
40	Risk factors for positive and negative COVID-19 tests: a cautious and in-depth analysis of UK biobank data (International Journal of Epidemiology)	Chadeau-Hyam, M., Bodinier, B., Elliott, J., Whitaker, M. D., Tzoulaki, I., Vermeulen, R., ... & Elliott, P. (2020). Risk factors for positive and negative COVID-19 tests: a cautious and in-depth analysis of UK biobank data. *International journal of epidemiology* , *49* (5), 1454-1467.	https://doi.org/10.1093/ije/dyaa134	M Chadeau-Hyam, B Bodinier, J Elliott	2020	7/3/2020	1	United Kingdom	488083 patients, 4509 CC	1					1	Logistic Regression	2	1	0	1	0	0	0		Comorbidities, Education, Employment, Incon	No
41	Coronavirus disease 2019 mortality: a multivariate ecological analysis in relation to ethnicity, population density, obesity, deprivation and pollution (Public Health)	Bray, I., Gibson, A., & White, J. (2020). Coronavirus disease 2019 mortality: a multivariate ecological analysis in relation to ethnicity, population density, obesity, deprivation and pollution. *Public health* , *185* , 261-263.	https://doi.org/10.1016/j.puhe.2020.06.056	I Bray, A Gibson, J White	2020	7/7/2020	1	England	310 Local Authorities		1					Multivariate Linear Regression	1	0	0	0	0	0	0		Index of Multiple Deprivation, Race, Overweig	No
42	Air Pollutants and SARS-CoV-2 in 33 European countries	Lembo, R., Landoni, G., Cianfanelli, L., & Frontera, A. (2021). Air pollutants and SARS-CoV-2 in 33 European countries. *Acta Bio Medica: Atenei Parmensis* , *92* (1).	https://10.23750/abm.v92i1.11155	R Lembo, G Landoni, L Cianfanelli	2021	2/16/2021	1	Austria, Belgium. Bulgaria, Croatia, Cyprus, Czec		1	1					Pearson Correlation	2	2	0	2	0	0	0	NH3-2, NMVOCs- 2	None	No
43	The Effects of Air Pollution, Sea Exposure and Altitude on COVID-19 Hospitalization Rates in Italy (International Journal of Environmental Research and Public Health)	Cascetta, E., Henke, I., & Di Francesco, L. (2021). The effects of air pollution, sea exposure and altitude on COVID-19 hospitalization rates in Italy. *International Journal of Environmental Research and Public Health* , *18* (2), 452.	https://doi.org/10.3390/ijerph18020452	E Cascetta, I Henke, L Di Francesco	2021	1/8/2021	1	Italy	107 provinces					1		Multivariate Linear Regression	2	0	0	0	0	0	0		Age, Location of Residence, Public Green Spa	No
44	The determinants of COVID-19 case fatality rate (CFR) in the Italian regions and provinces: An analysis of environmental, demographic, and healthcare factors (Science of the Total Environment)	Perone, G. (2021). The determinants of COVID-19 case fatality rate (CFR) in the Italian regions and provinces: An analysis of environmental, demographic, and healthcare factors. *Science of the Total Environment* , *755* , 142523.	https://doi.org/10.1016/j.scitotenv.2020.142523	G Perone	2020	9/24/2020	1	Italy	107 provinces				1			Multivariate Linear Regression	2	2	2	2	0	0	0		Temperature, Age, Urbanization, Hospital Bed	No
45	Spatial analysis of COVID-19 and traffic-related air pollution in Los Angeles (Environment International)	Lipsitt, J., Chan-Golston, A. M., Liu, J., Su, J., Zhu, Y., & Jerrett, M. (2021). Spatial analysis of COVID-19 and traffic-related air pollution in Los Angeles. *Environment International* .	https://doi.org/10.1016/j.envint.2021.106531	J Lipsitt, AM Chan-Golston, J Liu, J Su	2021	3/23/2021	1	United States	Los Angeles (348 neighbo	1	1		1			Poisson Mixed Model	0	0	0	2	0	0	0		Age, Income, Race, Comorbidities, Smoking	No

46	Risk for COVID-19 infection and death among Latinos in the United States: examining heterogeneity in transmission dynamics (Annals of Epidemiology)	Rodriguez-Diaz, C. E., Guilamo-Ramos, V., Mena, L., Hall, E., Honermann, B., Crowley, J. S., ... & Millett, G. A. (2020). Risk for COVID-19 infection and death among Latinos in the United States: examining heterogeneity in transmission dynamics. Annals of epidemiology, 52, 46-53.	https://doi.org/10.1016/j.annepidem.2020.07.007	CE Rodriguez-Diaz, V Guilamo-Ramos, L Mena	2020	7/23/2020	1	United States	3143 counties	1	1					Bayesian Negative Binomial Models	2	0	0	0	0	0	0		Age, Region, Latino, Comorbidities, Days Since	No
47	Relationship between COVID-19 infection rates and air pollution, geo-meteorological, and social parameters (Environmental Monitoring and Assessment)	Hassan, M., Bhuiyan, M. A. H., Tareq, F., Bodrud-Doza, M., Tanu, S. M., & Rabbani, K. A. (2021). Relationship between COVID-19 infection rates and air pollution, geo-meteorological, and social parameters. *Environmental Monitoring and Assessment*, *193*(1), 1-20.	https://doi.org/10.1007/s10661-020-08810-4	M Hassan, MAH Bhuiyan, F Tareq	2021	1/4/2021	1	Bangladesh	Dhaka (46 thanas)	1						Multivariate Regression	2	0	2	1	1	2	0	Aerosol Optical Thickness- 2, Water Vapor- 2	None	No
48	Machine learning reveals that prolonged exposure to air pollution is associated with SARS-CoV-2 mortality and infectivity in Italy (Environmental Pollution)	Gatti, R. C., Velichevskaya, A., Tateo, A., Amoroso, N., & Monaco, A. (2020). Machine learning reveals that prolonged exposure to air pollution is associated with SARS-CoV-2 mortality and infectivity in Italy. *Environmental Pollution*, *267*, 115471.	https://doi.org/10.1016/j.envpol.2020.115471	RC Gatti, A Velichevskaya, A Tateo, N Amoroso	2020	8/21/2020	1	Italy	20 regions	1	1					Random Forest	2	2	2	2	2	2	2	Benzene- 2	number of smokers, the level of obesity and d	Yes
49	Long-term Air Pollution Exposure Impact on COVID-19 Morbidity in China (Aerosol and Air Quality Research)	Wu, Y., Zhan, Q., & Zhao, Q. (2021). Long-term air pollution exposure impact on COVID-19 morbidity in China. *Aerosol and Air Quality Research*, *21*(1), 200413.	https://doi.org/10.4209/aaqr.2020.07.0413	Y Wu, Q Zhan, Q Zhao	2020	9/25/2020	1	China	326 prefectures		1					Negative Binomial Regression	2	2	2	2	1	1	0		Percent population with college degree, over	No
50	Long-term exposure to fine particulate matter and hospitalization in COVID-19 patients (Respiratory Medicine)	Mendy, A., Wu, X., Keller, J. L., Fassler, C. S., Apewokin, S., Mersha, T. B., ... & Pinney, S. M. (2021). Long-term exposure to fine particulate matter and hospitalization in COVID-19 patients. Respiratory medicine, 178, 106313.	https://doi.org/10.1016/j.rmed.2021.106313	A Mendy, X Wu, JL Keller, CS Fassler, S Apewokin	2021	1/26/2021	1	United States	Cincinnati					1		Logistic Regression	2	0	0	0	0	0	0		age and median household income used as co	Yes
51	Geographically varying relationships of COVID-19 mortality with different factors in India (Scientific Reports)	Middya, A. I., & Roy, S. (2021). Geographically varying relationships of COVID-19 mortality with different factors in India. *Scientific Reports*, *11*(1), 1-12.	https://doi.org/10.1038/s41598-021-86987-5	AI Middya, S Roy	2021	4/12/2021	1	India	more than 400 districts		1					Geographically Weighted Regression	2	0	0	0	0	0	0		District-level count of total number of househ	No
52	Effects of chronic exposure to ambient air pollutants on COVID-19 morbidity and mortality - A lesson from OECD countries (Environmental Research)	Barnett-Itzhaki, Z., & Levi, A. (2021). Effects of chronic exposure to ambient air pollutants on COVID-19 morbidity and mortality-A lesson from OECD countries. *Environmental research*, *195*, 110723.	https://doi.org/10.1016/j.envres.2021.110723	Z Barnett-Itzhaki, A Levi	2021	1/21/2021	1	Australia, Austria, Belgium, Canada, Chile, Czech		1	1					Multivariate Linear Regression	2	0	0	2	0	0	0		Numbers of physicians per 100,000 populatio	No
53	Ambient nitrogen dioxide pollution and spread ability of COVID-19 in Chinese cities (Ecotoxicology and Environmental Safety)	Yao, Y., Pan, J., Liu, Z., Meng, X., Wang, W., Kan, H., & Wang, W. (2021). Ambient nitrogen dioxide pollution and spreadability of COVID-19 in Chinese cities. *Ecotoxicology and environmental safety*, *208*, 111421.	https://doi.org/10.1016/j.ecoenv.2020.111421	Yao et al.,	2020	9/30/2020	2	China	11 cities in Hubei Province						1	Linear regression	0	0	0	2	0	0	0		None	No
54	Temporal association between particulate matter pollution and case fatality rate of COVID-19 in Wuhan Environmental Research)	Yao, Y., Pan, J., Liu, Z., Meng, X., Wang, W., Kan, H., & Wang, W. (2020). Temporal association between particulate matter pollution and case fatality rate of COVID-19 in Wuhan. Environmental research, 189, 109941.	https://doi.org/10.1016/j.envres.2020.109941	Yao et al.,	2020	7/15/2020	2	China	Wuhan				1			Multiple linear regression	2	2	0	0	0	0	0		Temperature, relative humidity	No
55	Effect of ambient air pollutants and meteorological variables on COVID-19 incidence (*Infection Control and Hospital Epidemiology*)	Jiang, Y., Wu, X. J., & Guan, Y. J. (2020). Effect of ambient air pollutants and meteorological variables on COVID-19 incidence. *Infection Control & Hospital Epidemiology*, *41*(9), 1011-1015.	https://doi.org/10.1017/ice.2020.222	Jiang et al.,	2020	5/11/2020	2	China	Wuhan, XiaoGan and Hu	1						Multivariate Poisson regression	2	1	1	1	1	1	0		Temperature, relative humidity, wind speed	No
56	Possible environmental effects on the spread of COVID-19 in China (*Science of the Total Environment*)	Xu, H., Yan, C., Fu, Q., Xiao, K., Yu, Y., Han, D., ... & Cheng, J. (2020). Possible environmental effects on the spread of COVID-19 in China. *Science of the Total Environment*, *731*, 139211.	https://doi.org/10.1016/j.scitotenv.2020.139211	Xu et al.,	2020	5/7/2020	2	China	33 cities	1						Multivariate Poisson regression	0	0	0	0	0	0	2		Temperature, relative humidity	No
57	Airborne particulate matter, population mobility and COVID-19: a multi-city study in China (BMC Public Health)	Wang, B., Liu, J., Li, Y., Fu, S., Xu, X., Li, L., ... & Zhang, K. (2020). Airborne particulate matter, population mobility and COVID-19: a multi-city study in China. *BMC public health*, *20*(1), 1-10.	https://doi.org/10.1186/s12889-020-09669-3	Wang et al.,	2020	10/21/2020	2	China	63 cities	1						Generalized additive models (GAM) with quasi-Poisson distribution for each city and meta analyses to aggregate results from each city	2	2	0	0	0	0	0		Ambient temperature, absolute humidity, migr	No
58	Air pollution and temperature are associated with increased COVID-19 incidence: a time series study (*International Journal of Infectious Diseases*)	Li, H., Xu, X. L., Dai, D. W., Huang, Z. Y., Ma, Z., & Guan, Y. J. (2020). Air pollution and temperature are associated with increased COVID-19 incidence: a time series study. *International journal of infectious diseases*, *97*, 278-282.	https://doi.org/10.1016/j.ijid.2020.05.076	Li et al.,	2020	6/2/2020	2	China	Wuhan and XiaoGan	1						Univariate linear regression	2	2	0	2	0	2	2		None	No
59	Outdoor PM2.5 concentration and rate of change in COVID-19 infection in provincial capital cities in China (Scientific Reports)	Han, Y., Lam, J. C., Li, V. O., Crowcroft, J., Fu, J., Downey, J., ... & Gilani, Z. (2021). Outdoor PM2. 5 concentration and rate of change in COVID-19 infection in provincial capital cities in China. *Scientific reports*, *11*(1), 1-12.	https://doi.org/10.1038/s41598-021-02523-5	Han et al.,	2021	12/1/2021	2	China	Wuhan	1						Multiple linear regression	2	0	0	0	0	0	0		Lockdown policy (binary), mobility, temperat	No
60	Correlation between climate indicators and COVID-19 pandemic in New York, USA (*Science of the Total Environment*)	Bashir, M. F., Ma, B., Komal, B., Bashir, M. A., Tan, D., & Bashir, M. (2020). Correlation between climate indicators and COVID-19 pandemic in New York, USA. *Science of The Total Environment*, *728*, 138835.	https://doi.org/10.1016/j.scitotenv.2020.138835	Bashir et al.,	2020	4/21/2020	2	United States	New York City	1	1					Kendall correlation coefficient and Spearman correlation coefficient	0	0	0	0	0	0	2		None	No
61	Association between short-term exposure to air pollution and COVID-19 infection: evidence from China (*Science of the Total Environment*)	Zhu, Y., Xie, J., Huang, F., & Cao, L. (2020). Association between short-term exposure to air pollution and COVID-19 infection: Evidence from China. *Science of the total environment*, *727*, 138704.	https://doi.org/10.1016/j.scitotenv.2020.138704	Zhu et al.,	2020	4/15/2020	2	China	120 cities	1						GAM with a Gaussian distribution	2	2	2	2	1	2	0		Temperature, relative humidity, air pressure, w	No
62	Air Pollution exposure and COVID-19: A look at mortality in Mexico City using individual-level data (Science of the Total Environment)	López-Feldman, A., Heres, D., & Marquez-Padilla, F. (2021). Air pollution exposure and COVID-19: a look at mortality in Mexico City using individual-level data. *Science of the Total Environment*, *756*, 143929.	https://doi.org/10.1016/j.scitotenv.2020.143929	Lopez-Feldman et al,	2020	11/26/2020	2	Mexico	71,620 individuals in the Mexico City Metropoli		1					Probit Model	2	0	0	0	0	0	0		Municipal-Level: population density, density	No
63	Assessing the Relationship between Ground levels of Ozone (O_3) and Nitrogen Dioxide (NO_2) and Coronavirus (COVID-19) in Milan, Italy (Science of the Total Environment)	Zoran, M. A., Savastru, R. S., Savastru, D. M., & Tautan, M. N. (2020). Assessing the relationship between ground levels of ozone (O3) and nitrogen dioxide (NO2) with coronavirus (COVID-19) in Milan, Italy. *Science of the Total Environment*, *740*, 140005.	https://doi.org/10.1016/j.scitotenv.2020.140005	Zoran et al,	2020	6/5/2020	2	Italy	Milan	1	1					Pearson Correlation Test	0	0	2	1	0	0	0		None	No
64	Assessing the relationship between surface levels of PM2.5 and PM10 particulate matter impact on COVID-19 in Milan, Italy (Science of the Total Environment)	Zoran, M. A., Savastru, R. S., Savastru, D. M., & Tautan, M. N. (2020). Assessing the relationship between surface levels of PM2. 5 and PM10 particulate matter impact on COVID-19 in Milan, Italy. *Science of the total environment*, *738*, 139825.	https://doi.org/10.1016/j.scitotenv.2020.139825	Zoran et al,	2020	6/2/2020	2	Italy	Milan	1	1					Pearson Correlation Test	2	2	0	0	0	0	0		None	No
65	The mediating effect of air quality on the association between human mobility and COVID-19 infection in China	Zhu, Y., Xie, J., Huang, F., & Cao, L. (2020). The mediating effect of air quality on the association between human mobility and COVID-19 infection in China. *Environmental research*, *189*, 109911.	https://doi.org/10.1016/j.envres.2020.109911	Y Zhu, J Xie, F Huang, L Cao	2020	7/9/2020	2	China	120 cities	1						Mediation Analysis	2	2	1	2	1	1	1		None	No
66	Impact of meteorological conditions and air pollution on COVID-19 pandemic transmission in Italy	Lolli, S., Chen, Y. C., Wang, S. H., & Vivone, G. (2020). Impact of meteorological conditions and air pollution on COVID-19 pandemic transmission in Italy. *Scientific reports*, *10*(1), 1-15.	https://doi.org/10.1038/s41598-020-73197-8	S Lolli, YC Chen, SH Wang, G Vivone	2020	10/1/2020	2	Italy	Milan, Florence, Trento					1		Spearman Correlation	2	0	0	2	0	0	0		None	No
67	Association of environmental and meteorological factors on the spread of COVID-19 in Victoria, Mexico, and air quality during the lockdown	Tello-Leal, E., & Macías-Hernández, B. A. (2021). Association of environmental and meteorological factors on the spread of COVID-19 in Victoria, Mexico, and air quality during the lockdown. *Environmental Research*, *196*, 110442.	https://doi.org/10.1016/j.envres.2020.110442	E Tello-Leal, BA Macías-Hernández	2020	11/11/2020	2	Mexico	Victoria	1						Spearman Correlation	2	2	0	0	0	2	0		None	No
68	Effects of air pollution on the potential transmission and mortality of COVID-19: A preliminary case-study in Tarragona Province (Catalonia, Spain)	Marquès, M., Rovira, J., Nadal, M., & Domingo, J. L. (2021). Effects of air pollution on the potential transmission and mortality of COVID-19: a preliminary case-study in Tarragona Province (Catalonia, Spain). *Environmental Research*, *192*, 110315.	https://doi.org/10.1016/j.envres.2020.110315	M Marquès, J Rovira, M Nadal, JL Domingo	2020	10/12/2020	2	Spain	Taragona Province	1						Pearson Correlation	0	1	2	2	0	0	0		None	No
69	Associations between air pollution and COVID-19 epidemic during quarantine period in China	Zhang, X., Tang, M., Guo, F., Wei, F., Yu, Z., Gao, K., ... & Chen, K. (2021). Associations between air pollution and COVID-19 epidemic during quarantine period in China. *Environmental Pollution*, *268*, 115897.	https://doi.org/10.1016/j.envpol.2020.115897	X Zhang, M Tang, F Guo, F Wei, Z Yu, K Gao, M Jin	2020	10/20/2020	2	China	235 cities	1						GAM	2	2	1	2	1	1	2		Temperature, dewpoint, lockdown, week, day	No
70	Short-Term Effects of Ambient Ozone, PM(2.5,) and Meteorological Factors on COVID-19 Confirmed Cases and Deaths in Queens, New York	Adhikari, A., & Yin, J. (2020). Short-term effects of ambient ozone, PM2. 5, and meteorological factors on COVID-19 confirmed cases and deaths in Queens, New York. *International journal of environmental research and public health*, *17*(11), 4047.	https://doi.org/10.3390/ijerph17114047	Adhikari et. al.,	2020	6/9/2020	2	United States	Queens County, New Yo	1	1					Negative Binomial Regression	1	0	2	0	0	0	0		Lagged outcome and day trend	No
71	Sustainable Ambient Environment to Prevent Future Outbreaks: How Ambient Environment Relates to COVID-19 Local Transmission in Lima, Peru	Kuo, T. C., Pacheco, A. M., Iswara, A. P., Dermawan, D., Andhikaputra, G., & Chiang Hsieh, L. H. (2020). Sustainable Ambient Environment to Prevent Future Outbreaks: How Ambient Environment Relates to COVID-19 Local Transmission in Lima, Peru. *Sustainability*, *12*(21), 9277.	https://doi.org/10.3390/su12219277	TC Kuo, AM Pacheco, AP Iswara, D Dermawan	2020	11/9/2020	2	Peru	Lima							Multivariate Regression	1	2	0	0	0	0	0		Temperature	No
72	Short-term exposure to ambient air pollution in association with COVID-19 of two clusters in South Korea	Hoang, T., Nguyen, T. Q., & Tran, T. T. A. (2021). Short -term exposure to ambient air pollution in association with COVID-19 of two clusters in South Korea. *Tropical Medicine & International Health*, *26*(4), 478-491.	https://doi.org/10.1111/tmi.13538	T Hoang, TQ Nguyen, TTA Tran	2020	12/15/2020	2	South Korea	Two major COVID-19 clu	1						Generalized Additive Model	2	2	1	2	2	2	0		Temperature, Wind Speed, Humidity, Air Pres	No
73	Correlations between Meteorological Indicators, Air Quality and the COVID-19 Pandemic in 12 Cities across China	Huang, H., Liang, X., Huang, J., Yuan, Z., Ouyang, H., Wei, Y., & Bai, X. (2020). Correlations between meteorological indicators, air quality and the COVID-19 pandemic in 12 cities across China. *Journal of Environmental Health Science and Engineering*, *18*(2), 1491-1498.	https://doi.org/10.1007/s40201-020-00564-y	H Huang, X Liang, J Huang, Z Yuan, H Ouyang	2020	10/15/2020	2	China	12 cities	1	1					Linear Regression Correlation	0	0	0	0	0	0	2		Temperature, Humidity, Precipitation	No
74	Effects of meteorological conditions and air pollution on COVID-19 transmission: evidence from 219 Chinese cities	Zhang, Z., Xue, T., & Jin, X. (2020). Effects of meteorological conditions and air pollution on COVID-19 transmission: Evidence from 219 Chinese cities. *Science of the total environment*, *741*, 140244.	https://doi.org/10.1016/j.scitotenv.2020.140244	Z Zhang, T Xue, X Jin	2020	6/16/2020	2	China	219 cities	1						Linear Regression	0	0	0	0	0	0	2		Temperature, Humidity, Wind Speed	No
75	The association between COVID-19 deaths and short-term ambient air pollution/meteorological condition exposure: a retrospective study from Wuhan, China (Air Quality, Atmosphere, and Health)	Jiang, Y., & Xu, J. (2021). The association between COVID-19 deaths and short-term ambient air pollution/meteorological condition exposure: a retrospective study from Wuhan, China. *Air Quality, Atmosphere & Health*, *14*(1), 1-5.	https://doi.org/10.1007/s11869-020-00906-7	Y Jiang, J Xu	2020	8/15/2020	2	China	Wuhan		1					Poisson Regression	2	1	1	1	1	1	0		Temperature and Humidity	No
76	Effects of climatological parameters on the outbreak spread of COVID-19 in highly affected regions of Spain (Environmental Science and Pollution Research)	Shahzad, K., Shahzad, U., Iqbal, N., Shahzad, F., & Fareed, Z. (2020). Effects of climatological parameters on the outbreak spread of COVID-19 in highly affected regions of Spain. *Environmental Science and Pollution Research*, *27*(31), 39657-39666.	https://doi.org/10.1007/s11356-020-10551-3	K Shahzad, U Shahzad, N Iqbal, F Shahzad	2020	8/22/2020	2	Spain	Castilla y Leon, Castilla-L	1						Panel Regression	2	0	0	0	0	0	0		Temperature	No
77	Environmental pollution and COVID-19 outbreak: insights from Germany (Air Quality, Atmosphere, and Health)	Bashir, M. F., Benghoul, M., Numan, U., Shakoor, A., Komal, B., Bashir, M. A., ... & Tan, D. (2020). Environmental pollution and COVID-19 outbreak: insights from Germany. *Air Quality, Atmosphere & Health*, *13*(11), 1385-1394.	https://doi.org/10.1007/s11869-020-00893-9	MF Bashir, M Benghoul, U Numan, A Shakoor	2020	10/10/2020	2	Germany		1	1					Coherence	2	2	2	2	0	0	0		None	No
78	Region-specific air pollutants and meteorological parameters influence COVID-19: A study from mainland China (Ecotoxicology and Environmental Safety)	Lin, S., Wei, D., Sun, Y., Chen, K., Yang, L., Liu, B., ... & Wu, S. (2020). Region-specific air pollutants and meteorological parameters influence COVID-19: A study from mainland China. *Ecotoxicology and environmental safety*, *204*, 111035.	https://doi.org/10.1016/j.ecoenv.2020.111035	S Lin, D Wei, Y Sun, K Chen, L Yang, B Liu	2020	8/5/2020	2	China	29 provinces			1				Correlation	0	0	0	1	0	2	0		None	No
79	The association between air pollution and COVID-19 related mortality in Santiago, Chile: A daily time series analysis (Environmental Research)	Dales, R., Blanco-Vidal, C., Romero-Meza, R., Schoen, S., Lukina, A., & Cakmak, S. (2021). The association between air pollution and COVID-19 related mortality in Santiago, Chile: a daily time series analysis. *Environmental research*, *198*, 111284.	https://doi.org/10.1016/j.envres.2021.111284	R Dales, C Blanco-Vidal, R Romero-Meza	2021	5/8/2021	2	Chile	Santiago		1					Poisson Mixed Model	0	0	0	0	0	0	1		Temperature, Humidity, and day of the week	No
80	Relationship between Weather Variables and New Daily COVID-19 Cases in Dhaka, Bangladesh (Sustainability)	Mofijur, M., Rizwanul Fattah, I. M., Saiful Islam, A. B. M., Uddin, M. N., Ashrafur Rahman, S. M., Chowdhury, M. A., ... & Uddin, M. (2020). Relationship between weather variables and new daily COVID-19 cases in Dhaka, Bangladesh. *Sustainability*, *12*(20), 8319.	https://doi.org/10.3390/su12208319	M Mofijur, IM Rizwanul Fattah, ABM Saiful Islam	2020	10/9/2020	2	Bangladesh	Dhaka	1						Correlation	0	0	0	0	0	0	1		None	No
81	Impact of climate and ambient air pollution on the epidemic growth during COVID-19 outbreak in Japan (Environmental Research)	Azuma, K., Kagi, N., Kim, H., & Hayashi, M. (2020). Impact of climate and ambient air pollution on the epidemic growth during COVID-19 outbreak in Japan. *Environmental research*, *190*, 110042.	https://doi.org/10.1016/j.envres.2020.110042	K Azuma, N Kagi, H Kim, M Hayashi	2020	8/12/2020	2	Japan	28 geographical areas	1						Mixed Model	2	0	1	1	0	0	0	suspended particulate matter- 2	Sex, Age, Income, Life Expectancy	No
82	Association between the New COVID-19 Cases and Air Pollution with Meteorological Elements in Nine Counties of New York State (International Journal of Environmental Research and Public Health)	Díaz-Avalos, C., Juan, P., Chaudhuri, S., Sáez, M., & Serra, L. (2020). Association between the New COVID-19 Cases and Air Pollution with Meteorological Elements in Nine Counties of New York State. *International Journal of Environmental Research and Public Health*, *17*(23), 9055.	https://doi.org/10.3390/ijerph17239055	C Díaz-Avalos, P Juan, S Chaudhuri, M Sáez	2020	12/4/2020	2	United States	New York State	1						Mixed Model	2	0	2	0	0	0	0		Temperature, Dewpoint, Humidity, Wind Spe	No
83	How do air pollution and meteorological parameters contribute to the spread of COVID-19 in Saudi Arabia? (Environmental Science Pollution Research)	Ben Maatoug, A., Triki, M. B., & Fazel, H. (2021). How do air pollution and meteorological parameters contribute to the spread of COVID-19 in Saudi Arabia?. *Environmental Science and Pollution Research*, *28*(32), 44132-44139.	https://doi.org/10.1007/s11356-021-13582-6	A Ben Maatoug, MB Triki, H Fazel	2021	4/12/2021	2	Saudi Arabia	3 cities (Riaydh, Jeddah a	1						Poisson and Binomial Negative Models	0	2	2	2	0	0	0		Temperature, Wind Speed	No
84	Effect of environmental pollution PM2.5, carbon monoxide, and ozone on the incidence and mortality due to SARS-CoV-2 infection in London, United Kingdom (Journal of King Saud University – Science)	Meo, S. A., Abukhalaf, A. A., Sami, W., & Hoang, T. D. (2021). Effect of environmental pollution PM2. 5, carbon monoxide, and ozone on the incidence and mortality due to SARS-CoV-2 infection in London, United Kingdom. *Journal of King Saud University-Science*, *33*(3), 101373.	https://doi.org/10.1016/j.jksus.2021.101373	SA Meo, AA Abukhalaf, W Sami, TD Hoang	2021	2/17/2021	2	United Kingdom	London	1	1					Poisson Regression Analysis	2	0	2	0	0	2	0		None	No
85	Effect of environmental pollutants PM-2.5, carbon monoxide, and ozone on the incidence and mortality of SARS-COV-2 infection in ten wildfire affected counties in California (Science of the Total Environment)	Meo, S. A., Abukhalaf, A. A., Alomar, A. A., Alessa, O. M., Sami W., & Klonoff, D. C. (2021). Effect of environmental pollutants PM-2.5, carbon monoxide, and ozone on the incidence and mortality of SARS-COV-2 infection in ten wildfire affected counties in California. *Science of the Total Environment*, *757*, 143948.	https://doi.org/10.1016/j.scitotenv.2020.143948	SA Meo, AA Abukhalaf, AA Alomar, OM Alessa	2020	11/25/2020	2	United States	California (ten counties)	1	1					Correlation	2	0	1	0	0	2	0		None	No
86	Impact of weather on COVID-19 transmission in south Asian countries: An application of the ARIMAX model (Science of the Total Environment)	Hossain, M. S., Ahmed, S., & Uddin, M. J. (2021). Impact of weather on COVID-19 transmission in south Asian countries: an application of the ARIMAX model. *Science of The Total Environment*, *761*, 143315.	https://doi.org/10.1016/j.scitotenv.2020.143315	MS Hossain, S Ahmed, MJ Uddin	2020	11/2/2020	2	Pakistan		1						ARIMAX	2	0	0	0	0	0	0		Temperature, Rainfall	No
87	Trade-offs between short-term mortality attributable to NO_2 and O_3 changes during the COVID-19 lockdown across major Spanish cities (Environmental Pollution)	Achebak, H., Petetin, H., Quijal-Zamorano, M., Bowdalo, D., García-Pando, C. P., & Ballester, J. (2021). Trade-offs between short-term mortality attributable to NO2 and O3 changes during the COVID-19 lockdown across major Spanish cities. *Environmental Pollution*, *286*, 117220.	https://doi.org/10.1016/j.envpol.2021.117220	H Achebak, H Petetin, M Quijal-Zamorano	2021	5/4/2021	2	Spain	47 cities		1					Quasi-Poisson regression model and mixed effects meta-analysis	0	0	2	2	0	0	0		Temperature, Humidity	No
88	Analysis of the Chemical and Physical Environmental Aspects that Promoted the Spread of SARS-CoV-2 in the Lombard Area (International Journal Environmental Research Public Health)	Dragone, R., Licciardi, G., Grasso, G., Del Gaudio, C., & Chanussot, J. (2021). Analysis of the chemical and physical environmental aspects that promoted the spread of SARS-CoV-2 in the Lombard area. *International Journal of environmental research and public health*, *18*(3), 1226.	https://doi.org/10.3390/ijerph18031226	R Dragone, G Licciardi, G Grasso	2021	1/29/2021	2	Italy	Lombardy Region: 12 pro	1						Spatial Correlation, Temporal Analysis	2	2	1	1	1	2	0	NH3- 2	Temperature, Humidity	No

89	Weather Conditions and COVID-19 Incidence in a Cold Climate: A Time-Series Study in Finland (Frontiers in Public Health)	Heibati, B., Wang, W., Ryti, N. R., Dominici, F., Ducatman, A., Zhang, Z., & Jaakkola, J. J. (2021). Weather conditions and covid-19 incidence in a cold climate: a time-series study in finland. Frontiers in public health , 974.	https://doi.org/10.3389/fpubh.2020.605128	B Heibati, W Wang, NRI Ryti, F Dominici	2021	2/25/2021	2	Finland		1						Quasi-Poisson Generalized Additive Model	0	1	0	1	0	0	0		Temperature, Humidity	No
90	The New York City COVID-19 Spread in the 2020 Spring: A Study on the Potential Role of Particulate Using Time Series Analysis and Machine Learning (Applied Sciences)	Mirri, S., Roccetti, M., & Delnevo, G. (2021). The new york city covid-19 spread in the 2020 spring: a study on the potential role of particulate using time series analysis and machine learning. Applied Sciences , 11(3), 1177.	https://doi.org/10.3390/app11031177	S Mirri, M Roccetti, G Delnevo	2021	1/27/2021	2	United States	New York City	1						Granger Causality	2	0	0	0	0	0	0		None	No
91	The ground-level ozone concentration is inversely correlated with the number of COVID-19 cases in Warsaw, Poland (Air Quality, Atmosphere, and Health)	Wiśniewski, O., Kozak, W., & Wiśniewski, M. (2021). The ground-level ozone concentration is inversely correlated with the number of COVID-19 cases in Warsaw, Poland. Air Quality, Atmosphere & Health , 14(8), 1169-1173.	https://doi.org/10.1007/s11869-021-01009-7	O Wiśniewski, W Kozak, M Wiśniewski	2021	4/8/2021	2	Poland	Warsaw	1						Correlation	0	0	1	0	0	0	0		None	No
92	Temporal and spatial analysis of COVID-19 transmission in China and its influencing factors (International Journal of Infectious Disease)	Wang, Q., Dong, W., Yang, K., Ren, Z., Huang, D., Zhang, P., & Wang, J. (2021). Temporal and spatial analysis of COVID-19 transmission in China and its influencing factors. International Journal of Infectious Diseases , 105, 675-685.	https://doi.org/10.1016/j.ijid.2021.03.014	Q Wang, W Dong, K Yang, Z Ren, D Huang	2021	3/10/2021	2	China	prefecture-level cities	1						Correlation	2	2	0	0	2	2	2		None	No
93	Spatial sensitivity analysis of COVID-19 infections concerning the satellite-based four air pollutants levels (International Journal of Environmental Science and Technology)	Bigdeli, M., Taheri, M., & Mohammadian, A. (2021). Spatial sensitivity analysis of COVID-19 infections concerning the satellite-based four air pollutants levels. International Journal of Environmental Science and Technology, 18(3), 751-760.	https://doi.org/10.1007/s13762-020-03112-1	M Bigdeli, M Taheri, A Mohammadian	2021	1/9/2021	2	Iran	31 provinces	1						Multivariate Linear Regression	0	0	2	2	2	2	0		None	No
94	Spatial and temporal trends in social vulnerability and COVID-19 incidence and death rates in the United States (PLOS One)	Neelon, B., Mutiso, F., Mueller, N. T., Pearce, J. L., & Benjamin-Neelon, S. E. (2021). Spatial and temporal trends in social vulnerability and COVID-19 incidence and death rates in the United States. Plos one , 16(3), e0248702.	https://doi.org/10.1371/journal.pone.0248702	B Neelon, F Mutiso, NT Mueller, JL Pearce	2021	3/24/2021	2	United States	3142 counties	1	1					Bayesian hierarchical negative binomial models	2	0	0	0	0	0	0		Sex, Rural, Smoking, Health, Physicians, Tem	No
95	Significance between air pollutants, meteorological factors, and COVID-19 infections: probable evidences in India (Environmental Science and Pollution Research)	Sahoo, M. M. (2021). Significance between air pollutants, meteorological factors, and COVID-19 infections: probable evidences in India. Environmental Science and Pollution Research, 28(30), 40474-40495.	https://doi.org/10.1007/s11356-021-12709-z	MM Sahoo	2021	2/27/2021	2	India	8 states	1						Generalized Additive Model	2	2	0	2	1	0	0		Temperature, Humidity, Rainfall, Air pressure	No
96	PM 10 correlates with COVID-19 infections 15 days later in Arequipa, Peru (Environmental Science and Pollution Research)	Wannaz, E. D., Larrea Valdivia, A. E., Reyes Larico, J. A., Salcedo Peña, J., & Valenzuela Huillca, C. (2021). PM10 correlates with COVID-19 infections 15 days later in Arequipa, Peru. Environmental Science and Pollution Research , 28(29), 39648-39654.	https://doi.org/10.1007/s11356-021-13408-5	ED Wannaz, AE Larrea Valdivia	2021	3/24/2021	2	Peru	Arequipa	1						Correlation	1	2	0	0	0	0	0		None	No
97	Particulate Matter Short-Term Exposition, Mobility Trips and COVID-19 Diffusion: A Correlation Analyses for the Italian Case Study at Urban Scale (Sustainability)	Carteni, A., Cascetta, F., Di Francesco, L., & Palermo, F. (2021). Particulate Matter Short-Term Exposition, Mobility Trips and COVID-19 Diffusion: A Correlation Analyses for the Italian Case Study at Urban Scale. Sustainability , 13(8), 4553.	https://doi.org/10.3390/su13084553	A Carteni, F Cascetta, L Di Francesco, F Palermo	2021	4/20/2021	2	Italy	13 cities	1						Correlation	2	2	0	0	0	0	0		None	No
98	Pandemic meets pollution: Poor air quality increases deaths by COVID-19 (Journal of Environmental Economics and Management)	Isphording, I. E., & Pestel, N. (2021). Pandemic meets pollution: poor air quality increases deaths by COVID-19. Journal of Environmental Economics and Management , 108, 102448.	https://doi.org/10.1016/j.jeem.2021.102448	IE Isphording, N Pestel	2021	4/8/2021	2	Germany	294 counties	1	1					Multivariate Linear Regression	2	2	1	0	0	0	0		Precipitation, Wind Speed, Temperature	No
99	Meteorological factors, COVID-19 cases, and deaths in top 10 most affected countries: an econometric investigation (Environmental Science and Pollution Research International)	Sharma, G. D., Bansal, S., Yadav, A., Jain, M., & Garg, I. (2021). Meteorological factors, COVID-19 cases, and deaths in top 10 most affected countries: an econometric investigation. Environmental Science and Pollution Research, 28(22), 28624-28639.	https://doi.org/10.1007/s11356-021-12668-5	GD Sharma, S Bansal, A Yadav, M Jain	2021	2/8/2021	2	United States, India, Brazil, Russia, Spain, Unite		1	1					Panel Data Regression	2	0	0	0	0	0	0		Air Pressure, Humidity, Temperature, Wind S	No
100	Lag Effects of Ozone, PM2.5, and Meteorological Factors on COVID-19 New Cases at the Disease Epicenter in Queens, New York (Atmosphere)	Adhikari, A., & Yin, J. (2021). Lag Effects of Ozone, PM2. 5, and Meteorological Factors on COVID-19 New Cases at the Disease Epicenter in Queens, New York. Atmosphere, 12(3), 357.	https://doi.org/10.3390/atmos12030357	A Adhikari, J Yin	2021	3/9/2021	2	United States	New York City (Queens	1						Negative Binomial Regression	2	0	2	0	0	0	0		Temperature, Cloud Cover, Humidity	No
101	Investigating the Effects of Meteorological Parameters on COVID-19: Case Study of New Jersey, United States (Environmental Research)	Doğan, B., Jebli, M. B., Shahzad, K., Farooq, T. H., & Shahzad, U. (2020). Investigating the effects of meteorological parameters on COVID-19: case study of New Jersey, United States. Environmental Research , 191, 110148.	https://doi.org/10.1016/j.envres.2020.110148	B Doğan, MB Jebli, K Shahzad, TH Farooq	2020	8/30/2020	2	United States	New Jersey	1						Correlation	2	0	0	0	0	0	2		Temperature, Humidity	No
102	Influence of air pollution and meteorological factors on the spread of COVID-19 in the Bangkok Metropolitan Region and air quality during the outbreak (Environmental Research)	Sangkham, S., Thongtip, S., & Vongruang, P. (2021). Influence of air pollution and meteorological factors on the spread of COVID-19 in the Bangkok Metropolitan Region and air quality during the outbreak. Environmental Research , 197, 111104.	https://doi.org/10.1016/j.envres.2021.111104	S Sangkham, S Thongtip, P Vongruang	2021	3/30/2021	2	Thailand	Bangkok	1						Correlation	1	1	1	1	1	1	1		None	No
103	Econometric analysis of COVID-19 cases, deaths, and meteorological factors in South Asia (Environmental Science and Pollution Research International)	Jain, M., Sharma, G. D., Goyal, M., Kaushal, R., & Sethi, M. (2021). Econometric analysis of COVID-19 cases, deaths, and meteorological factors in South Asia. Environmental Science and Pollution Research , 28(22), 28518-28534.	https://doi.org/10.1007/s11356-021-12613-6	M Jain, GD Sharma, M Goyal, R Kaushal	2021	2/4/2021	2	Bangladesh, India, Nepal, Pakistan, Sri Lanka		1	1					Geographically Weighted Regression	1	0	0	0	0	0	0		Temperature, Wind Speed, Air Pressure, Hum	No
104	Dynamic model to predict the association between air quality, COVID-19 cases, and level of lockdown (Environmental Pollution)	Tadano, Y. S., Potgieter-Vermaak, S., Kachba, Y. R., Chiroli, D. M., Casacio, L., Santos-Silva, J. C., ... & Godoi, R. H. (2021). Dynamic model to predict the association between air quality, COVID-19 cases, and level of lockdown. Environmental Pollution , 268, 115920.	https://doi.org/10.1016/j.envpol.2020.115920	YS Tadano, S Potgieter-Vermaak, YR Kachba	2020	10/29/2020	2	Brazil	Sao Paulo	1						Artificial Neural Networks	2	2	2	2	0	2	0		Temperature, Humidity, Wind Speed, Global S	No
105	Do air pollutants as well as meteorological factors impact Corona Virus Disease 2019 (COVID-19)? Evidence from China based on the geographical perspective (Environmental Science and Pollution Research)	Pei, L., Wang, X., Guo, B., Guo, H., & Yu, Y. (2021). Do air pollutants as well as meteorological factors impact Corona Virus Disease 2019 (COVID-19)? Evidence from China based on the geographical perspective. Environmental Science and Pollution Research , 28(27), 35584-35596.	https://doi.org/10.1007/s11356-021-12934-6	L Pei, X Wang, B Guo, H Guo, Y Yu	2021	3/5/2021	2	China	325 cities	1						Geographically Weighted Regression	2	2	0	1	1	1	2		Temperature, Wind Speed, Humidity, Precipit	No
106	COVID-19 and air pollution in Vienna—a time series approach	Moshammer, H., Poteser, M., & Hutter, H. P. (2021). COVID-19 and air pollution in Vienna—a time series approach. Wiener Klinische Wochenschrift , 133(17), 951-957.	https://doi.org/10.1007/s00508-021-01881-4	Moshammer, H., Poteser, M. & Hutter, HP.	2021	5/6/2021	2	Austria	Vienna	1	1					Multivariate Linear Regression	0	2	0	2	0	0	0		Temperature, Day of the Week	No
107	Ambient air pollution, meteorology, and COVID-19 infection in Korea (Journal of Medical Virology)	Hoang, T., & Tran, T. T. A. (2021). Ambient air pollution, meteorology, and COVID-19 infection in Korea. Journal of medical virology , 93(2), 878-885.	https://doi.org/10.1002/jmv.26325	T Hoang, TTA Tran	2020	7/21/2020	2	Korea		1						Generalized Additive Model	2	1	1	2	2	2	0		Temperature, Wind Speed, Humidity, Air Pres	No
108	Association between exposure to airborne pollutants and COVID-19 in Los Angeles, United States with ensemble-based dynamic emission model (Environmental Research)	Gujral, H., & Sinha, A. (2021). Association between exposure to airborne pollutants and COVID-19 in Los Angeles, United States with ensemble-based dynamic emission model. Environmental research , 194, 110704.	https://doi.org/10.1016/j.envres.2020.110704	H Gujral, A Sinha	2021	1/5/2021	2	United States	Los Angeles	1	1					Generalized Additive Model	1	1	1	0	0	0	0		Air Pressure, Temperature, Humidity, Wind S	No
109	The interactive effects of ambient air pollutants-meteorological factors on confirmed cases of COVID-19 in 120 Chinese cities (Environmental Science and Pollution Research)	Zhou, J., Qin, L., Meng, X., & Liu, N. (2021). The interactive effects of ambient air pollutants-meteorological factors on confirmed cases of COVID-19 in 120 Chinese cities. Environmental Science and Pollution Research , 28(21), 27056-27066.	https://doi.org/10.1007/s11356-021-12648-9	Zhou et al.,	2021	1/27/2021	3	China	120 cities	1						Negative binomial regression	2	0	2	1	1	2	0		Average temperature range, diurnal temperatu	No
110	Associations between ambient air pollutants exposure and case fatality rate of COVID-19: a multi-city ecological study in China (medRxiv)	Zhang, T., Zhao, G., Luo, L., Li, Y., & Shi, W. (2020). Associations between ambient air pollutants exposure and case fatality rate of COVID-19: A multi-city ecological study in China. medRxiv .	https://doi.org/10.1101/2020.05.06.20088682	Zhang et al.,	2020	5/10/2020	3	China	24 provinces and 13 main cities in Hubei Province				1			Multiple linear regression	2	2	0	2	0	0	0		Other Pollutants	No
111	Ambient nitrogen dioxide pollution and spread ability of COVID-19 in Chinese cities (Ecotoxicology and Environmental Safety)	Yao, Y., Pan, J., Liu, Z., Meng, X., Wang, W., Kan, H., & Wang, W. (2021). Ambient nitrogen dioxide pollution and spreadability of COVID-19 in Chinese cities. Ecotoxicology and environmental safety , 208, 111421.	https://doi.org/10.1016/j.ecoenv.2020.111421	Yao et al.,	2020	9/30/2020	3	China	63 cities						1	Multiple linear regression	0	0	0	2	0	0	0		Temperature and humidity	No
112	Severe air pollution links to higher mortality in COVID-19 patients: the "double-hit" hypothesis (Journal of Infectious Diseases)	Frontera, A., Cianfanelli, L., Vlachos, K., Landoni, G., & Cremona, G. (2020). Severe air pollution links to higher mortality in COVID-19 patients: The "double-hit" hypothesis. Journal of Infection , 81(2), 255-259.	https://doi.org/10.1016/j.jinf.2020.05.031	Frontera et al.,	2020	5/21/2020	3	Italy	21 regions	1	1			1		Univariate linear regression	2	0	0	0	0	0	0		None	No
113	Association of particulate matter pollution and case fatality rate of COVID-19 in 49 Chinese cities (The Science of the Total Environment)	Yao, Y., Pan, J., Wang, W., Liu, Z., Kan, H., Qiu, Y., ... & Wang, W. (2020). Association of particulate matter pollution and case fatality rate of COVID-19 in 49 Chinese cities. Science of the Total Environment , 741, 140396.	https://doi.org/10.1016/j.scitotenv.2020.140396	Yao et al.,	2020	6/20/2020	3	China	49 cities				1			Multiple linear regression	2	2	0	0	0	0	0		Temperature, relative humidity, gross domesti	No
114	Spatial-temporal variations in atmospheric factors contribute to SARS-CoV-2 outbreak (Viruses)	Fronza, R., Lusic, M., Schmidt, M., & Lucic, B. (2020). Spatial–temporal variations in atmospheric factors contribute to SARS-CoV-2 outbreak. Viruses , 12(6), 588.	https://doi.org/10.3390/v12060588	Fronza et al.,	2020	5/28/2020	3	France, Germany, Spa	47 regional capitals, 107	1					1	Spearman correlation and an Artificial Neural Network (ANN) to predict epidemic escalation	2	2	2	0	0	0	0	NH3- 2	Temperature, dew point temperature, wind spe	Yes (https://github.com/COVID19Upcome/Europe)
115	COVID-19 pandemic in relation to levels of pollution with $PM_{2.5}$ and ambient salinity. an environmental Wake-up Call (medRxiv)	Baron, Y. M. (2020). Covid-19 Pandemic in relation to levels of Pollution with PM2. 5 and Ambient Salinity–An Environmental Wake-up Call. medRxiv .	https://doi.org/10.1101/2020.05.03.20087056	Baron et al.,	2020	5/9/2020	3	China, France, Spain,	7 cities with high numbers of COVID-19 cases, including Wuhan, Qom, Bergamo, Paris, Madrid, London, and New York						1	Unspecified	2	0	0	0	0	0	0		None	No
116	Potential role of particulate matter in the spreading of COVID-19 in Northern Italy: first observational study based on initial epidemic diffusion (BMJ Open)	Setti, L., Passarini, F., De Gennaro, G., Barbieri, P., Licen, S., Perrone, M. G., ... & Miani, A. (2020). Potential role of particulate matter in the spreading of COVID-19 in Northern Italy: first observational study based on initial epidemic diffusion. BMJ open , 10(9), e039338.	https://doi.org/10.1136/bmjopen-2020-039338	Setti et al.,	2020	9/24/2020	3	Italy	110 provinces	1						Multivariate binomial regression	0	2	0	0	0	0	0		Population density, number of people traveling	No
117	Assessing Correlations between Short-Term Exposure to Atmospheric Pollutants and COVID-19 Spread in All Italian Territorial Areas	Accarino, G., Lorenzetti, S., & Aloisio, G. (2021). Assessing correlations between short-term exposure to atmospheric pollutants and COVID-19 spread in all Italian territorial areas. Environmental Pollution , 268, 115714.	https://doi.org/10.1016/j.envpol.2020.115714	Accarino et al,	2020	10/15/2020	3	Italy	107 territorial areas	1	1				1	Spearman Correlation	2	2	0	2	0	0	0		None	No
118	Association of COVID-19 distribution with air quality, sociodemographic factors, and comorbidities: an ecological study of US states	Sarmadi, M., Moghanddam, V. K., Dickerson, A. S., & Martelletti, L. (2021). Association of COVID-19 distribution with air quality, sociodemographic factors, and comorbidities: an ecological study of US states. Air Quality, Atmosphere & Health , 14(4), 455-465.	https://doi.org/10.1007/s11869-020-00949-w	Sarmadi et al,	2020	10/14/2020	3	United States	50 states plus District of C	1	1		1			Multivariate linear regression	0	0	1	2	0	1	0	SO4-1	Race, Median Age, Comorbidities	No
119	Evidence-Based Considerations Exploring Relations between SARS-CoV-2 Pandemic and Air Pollution: Involvement of PM2.5-Mediated Up-Regulation of the Viral Receptor ACE-2 (International Journal of Environmental Research and Public Health)	Borro, M., Di Girolamo, P., Gentile, G., De Luca, O., Preissner, R., Marcolongo, A., ... & Simmaco, M. (2020). Evidence-based considerations exploring relations between SARS-CoV-2 pandemic and air pollution: involvement of PM2. 5-mediated up-regulation of the viral receptor ACE-2. International journal of environmental research and public health , 17(15), 5573.	https://doi.org/10.3390/ijerph17155573	M Borro, P Di Girolamo, G Gentile, O De Luca	2020	8/3/2020	3	Italy	110 Provinces	1	1		1			Correlation	2	0	0	0	0	0	0		None	No
120	Effects of particulate matter exposure on the transmissibility and case fatality rate of COVID-19: A Nationwide Ecological Study in China (Journal of Travel Medicine)	Ran, J., Zhao, S., Han, L., Qiu, Y., Cao, P., Yang, Z., ... & He, D. (2020). Effects of particulate matter exposure on the transmissibility and case fatality rate of COVID-19: A Nationwide Ecological Study in China. Journal of Travel Medicine , 27(6), taaa133.	https://doi.org/10.1093/jtm/taaa133	J Ran, S Zhao, L Han, Y Qiu, P Cao	2020	8/7/2020	3	China	154 Cities			1	1			Multivariate linear regression	2	2	0	0	0	0	0		None	No
121	The underlying factors of the COVID-19 spatially uneven spread. Initial evidence from regions in nine EU countries (Regional Science Policy and Practice)	Kapitsinis, N. (2020). The underlying factors of the COVID-19 spatially uneven spread. Initial evidence from regions in nine EU countries. Regional Science Policy & Practice , 12(6), 1027-1045.	https://doi.org/10.1111/rsp3.12340	N Kapitsinis	2020	9/3/2020	3	Germany, France, Gre	119 EU regions		1					Multivariate linear regression	0	2	0	0	0	0	0		Exports, Motor Way Density, Tourism, Air Tr	No
122	Effects of Demographic and Weather Parameters on COVID-19 Basic Reproduction Number (Frontiers of Ecology and Evolution)	Salom, I., Rodic, A., Milicevic, O., Zigic, D., Djordjevic, M., & Djordjevic, M. (2021). Effects of demographic and weather parameters on COVID-19 basic reproduction number. Frontiers in Ecology and Evolution , 8, 524.	https://doi.org/10.3389/fevo.2020.617841	I Salom, A Rodic, O Milicevic, D Zigic	2021	1/25/2021	3	118 countries							1	Correlation	1	1	0	2	2	1	0		None	No
123	COVID-19 Infection and Mortality: Association with PM2.5 Concentration and Population Density—An Exploratory Study	Yu, X., Wong, M. S., Kwan, M. P., Nichol, J. E., Zhu, R., Heo, J., ... & Kan, Z. (2021). COVID-19 infection and mortality: Association with PM2. 5 concentration and population density—An exploratory study. ISPRS International Journal of Geo-Information, 10(3), 123.	https://doi.org/10.3390/ijgi10030123	Yu et al.	2021	3/1/2021	1	251 countries		1	1					Geographically Weighted Regression	2	0	0	0	0	0	0		GDP per Capita, Smoking Rate, Hospital Beds	No
124	COVID-19 incidence and mortality in Lombardy, Italy: An ecological study on the role of air pollution, meteorological factors, demographic and socioeconomic variables	De Angelis, E., Renzetti, S., Volta, M., Donato, F., Calza, S., Placidi, D., ... & Rota, M. (2021). COVID-19 incidence and mortality in Lombardy, Italy: an ecological study on the role of air pollution, meteorological factors, demographic and socioeconomic variables. Environmental research, 195, 110777.	https://doi.org/10.1016/j.envres.2021.110777	Angelis et al.	2021	1/22/2021	1	Italy	1439 municipalities in Lo	1	1					Negative Binomial Mixed Regression Models	2	2	0	1	0	0	0		Population Density, Sex Ratio, Proportion of I	No
125	COVID-19 higher mortality in Chinese regions with chronic exposure to lower air quality	Pansini, R., & Fornacca, D. (2021). COVID-19 higher mortality in Chinese regions with chronic exposure to lower air quality. Frontiers in public health, 8, 999.	https://doi.org/10.3389/fpubh.2020.597753	R Pansini, D Fornacca	2021	1/22/2021	1	China	333 prefectures	1	1		1			Correlation	2	2	2	2	2	2	0	HCHO-2, Aerosol- 2	None	No
126	Community Risk Factors in the COVID-19 Incidence and Mortality in Catalonia (Spain). A Population-Based Study	Zaldo-Aubanell, Q., Campillo i Lopez, F., Bach, A., Serra, I., Olivet-Vila, J., Saez, M., ... & Maneja, R. (2021). Community risk factors in the COVID-19 incidence and mortality in Catalonia (Spain). A population-based study. International journal of environmental research and public health, 18(7), 3768.	https://doi.org/10.3390/ijerph18073768	Zaldo-Aubaneli et al.	2021	1/25/2021	1	Spain	372 Basic Health Areas	1	1					Logistic Regression	0	2	0	2	0	0	0		Sex, SES, Age, Comorbidities, Industry	No
127	Community factors and excess mortality in first wave of the COVID-19 pandemic in England	Davies, B., Parkes, B. L., Bennett, J., Fecht, D., Blangiardo, M., Ezzati, M., & Elliott, P. (2021). Community factors and excess mortality in first wave of the COVID-19 pandemic in England. Nature communications , 12(1), 1-9.	https://doi.org/10.1038/s41467-021-23935-x	Davies et al.	2021	6/18/2021	1	England	6791 MSOAs						1	Bayesian Spatial Model	1	0	0	1	0	0	0		Population Density, Income Deprivation, Ethn	Yes (SmallAreaHealthStatisticsUnit/SAHSU-COVID-19)
128	Can particulate matter be identified as the primary cause of the rapid spread of CoViD-19 in some areas of Northern Italy?	Collivignarelli, M. C., Abbà, A., Caccamo, F. M., Bertanza, G., Pedrazzani, R., Baldi, M., ... & Carnevale Miino, M. (2021). Can particulate matter be identified as the primary cause of the rapid spread of CoViD-19 in some areas of Northern Italy?. Environmental Science and Pollution Research, 28(25), 33120-33132.	https://doi.org/10.1007/s11356-021-12735-x	Collivignarelli et al.	2021	2/26/2021	2	Italy	41 cities	1						Correlation	2	1	0	0	0	0	0		None	No
129	Associations between mortality from COVID-19 in two Italian regions and outdoor air pollution as assessed through tropospheric nitrogen dioxide	Filippini, T., Rothman, K. J., Cocchio, S., Narne, E., Mantoan, D., Saia, M., ... & Vinceti, M. (2021). Associations between mortality from COVID-19 in two Italian regions and outdoor air pollution as assessed through tropospheric nitrogen dioxide. Science of the Total Environment, 760, 143355.	https://doi.org/10.1016/j.scitotenv.2020.143355	Filippini et al.	2020	11/4/2020	3	Italy	16 provinces		1					Negative Binomial Regression	0	0	0	2	0	0	0		Population Density, Aging Index, Mobility, T	No
130	Association of air pollution and meteorological variables with COVID-19 incidence: Evidence from five megacities in India	Kolluru, S. S. R., Patra, A. K., & Nagendra, S. S. (2021). Association of air pollution and meteorological variables with COVID-19 incidence: Evidence from five megacities in India. Environmental Research, 195, 110854.	https://doi.org/10.1016/j.envres.2021.110854	Kolluru et al.	2021	2/6/2021	2	India	5 cities	1	1					Correlation	2	2	1	0	0	2	2		None	No
131	Association between environmental factors and COVID-19 in Shanghai, China	Ma, Y., Cheng, B., Shen, J., Wang, H., Feng, F., Zhang, Y., & Jiao, H. (2021). Association between environmental factors and COVID-19 in Shanghai, China. Environmental Science and Pollution Research, 28(33), 45087-45095.	https://doi.org/10.1007/s11356-021-13834-5	Ma et al.	2021	4/15/2021	2	China	Shanghai	1						Generalized Additive Model	2	2	1	2	2	0	2		Temperature, Humidity, Air Pressure, Wind S	No

132	Association between coronavirus disease 2019 (COVID-19) and long-term exposure to air pollution: Evidence from the first epidemic wave in China	Zheng, P., Chen, Z., Liu, Y., Song, H., Wu, C. H., Li, B., ... & Jia, G. (2021). Association between coronavirus disease 2019 (COVID-19) and long-term exposure to air pollution: Evidence from the first epidemic wave in China. Environmental Pollution, 276, 116682.	https://doi.org/10.1016/j.envpol.2021.116682	Zheng et al.	2021	2/8/2021	1	China	324 cities	1						Poisson Regression	2	2	1	2	1	1	0		Mobility, GDP, Smoking, Population Greater	No
133	Association between air quality, meteorological factors and COVID-19 infection case numbers	Lorenzo, J. S. L., San Tam, W. W., & Seow, W. J. (2021). Association between air quality, meteorological factors and COVID-19 infection case numbers. Environmental Research, 197, 111024.	https://doi.org/10.1016/j.envres.2021.111024	Lorenzo et al.	2021	3/17/2021	2	Singapore		1						Poisson Regression	2	1	1	2	1	1	0		Humidity, Day of the Week, Precipitiation	No
134	Ambient PM2.5 exposure and rapid spread of COVID-19 in the United States	Chakrabarty, R. K., Beeler, P., Liu, P., Goswami, S., Harvey, R. D., Pervez, S., ... & Martin, R. V. (2021). Ambient PM2. 5 exposure and rapid spread of COVID-19 in the United States. Science of the Total Environment, 760, 143391.	https://doi.org/10.1016/j.scitotenv.2020.143391	Chakrabarty et al.	2020	11/9/2020	1	United States	50 states plus District of Columbia			1				Generalized Additive Model	2	0	0	0	0	0	0		Population, Age Structure, COVID-19 tests, H	Yes (https://github.com/pliu1991/COVID19PM25_supporting_data_and_files)
135	Air Pollution and COVID-19 Mortality in New York City	Kim, H., & Bell, M. L. (2021). Air pollution and COVID-19 mortality in New York City. American Journal of Respiratory and Critical Care Medicine, 204(1), 97-99.	https://doi.org/10.1164/rccm.202010-3844LE	H Kim, M Bell	2021	5/3/2021	1	United States	New York City (177 Neigborhoods)		1					Poisson Regression	2	0	2	0	0	0	0		Population, Race, Sex Ratio, Population, Hous	No
136	Global effect of city-to-city air pollution, health conditions, climatic & socio-economic factors on COVID-19 pandemic	Sarkodie, S. A., & Owusu, P. A. (2021). Global effect of city-to-city air pollution, health conditions, climatic & socio-economic factors on COVID-19 pandemic. Science of the Total Environment, 778, 146394.	https://doi.org/10.1016/j.scitotenv.2021.146394	S Sarkodie, P Owusu	2021	3/12/2021	2	615 Cities across 6 Continents (Many Countries)		1	1					Multiple Linear Regression	2	0	2	2	0	0	0		Temperature, Pressure, Humidity, Wind Speed	No
137	Impact of a long-term air pollution exposure on the case fatality rate of COVID-19 patients-A multicity study	Hou, C. K., Qin, Y. F., Wang, G., Liu, Q. L., Yang, X. Y., & Wang, H. (2021). Impact of a long-term air pollution exposure on the case fatality rate of COVID-19 patients—A multicity study. Journal of medical virology, 93(5), 2938-2946.	https://doi.org/10.1002/jmv.26807	Hou et al.	2021	1/20/2021	1	China	14 cities				1			Correlation	2	2	1	1	1	0	2		Population, Race, Sex Ratio, Population, Hous	No
138	Associations between COVID-19 Incidence Rates and the Exposure to PM2.5 and NO2: A Nationwide Observational Study in Italy	Fiasca, F., Minelli, M., Maio, D., Minelli, M., Vergallo, I., Necozione, S., & Mattei, A. (2020). Associations between COVID-19 incidence rates and the exposure to PM2. 5 and NO2: A nationwide observational study in Italy. International Journal of Environmental Research and Public Health, 17(24), 9318.	https://doi.org/10.3390/ijerph17249318	Fiasca et al.	2020	12/14/2020	1	Italy	62 Provinces	1						Multiple Linear Regression	2	0	0	2	0	0	0		Population Density, Elderly Population	No
139	Containment measures limit environmental effects on COVID-19 early outbreak dynamics	Ficetola, G. F., & Rubolini, D. (2021). Containment measures limit environmental effects on COVID-19 early outbreak dynamics. Science of The Total Environment, 761, 144432.	https://doi.org/10.1016/j.scitotenv.2020.144432	G Ficetola, D Rubolini	2020	12/16/2020	1	586 countries/regions		1						Linear Mixed Model	2	0	0	0	0	0	0		Population, Health Expenditure, Elderly Popul	No
