# Supplementary material for: A Literature Review of the Effects of Air Pollution on COVID-19 Health Outcomes Worldwide: Statistical Challenges and Data Visualization
Source: Annu Rev Public Health. Author manuscript; Available in PMC 2024 Nov 15. (PMC11567163; doi:10.1146/annurev-publhealth-071521-120424)
Supplement: supplemental material_b [file NIHMS2031411-supplement-supplemental_material_b.pdf]

Supplemental Material: Annu. Rev. Public Health 2023. 44:1-20
https://doi.org/10.1146/annurev-publhealth-071521-120424
A Literature Review of the Effects of Air Pollution on COVID-19 Health Outcomes Worldwide: Statistical Challenges and Data Visualization
Bhaskar et al.

Supplemental Table 1. Search Queries for All Relevant Databases (as of May 21, 2021)

PUBMED
(coronavirus OR “corona virus” OR coronavirinae OR coronaviridae OR betacoronavirus OR covid19 OR “covid 19” OR nCoV OR “CoV 2” OR CoV2 OR sarscov2 OR 2019nCoV OR “novel CoV” OR “wuhan virus” OR novel coronavirus[tiab] OR 2019-ncov[tiab] OR coronavirus[tiab] OR coronavirus disease-9[tiab] OR coronavirus*[tiab] OR coronovirus*[tiab] OR 2019-nCoV[tiab] OR COVID-19[tiab] OR Covid[tiab] OR ncov[tiab] OR SARS-CoV-2[tiab] OR SARSCov19[tiab] OR SARS-Covid[tiab] OR ((wuhan OR hubei OR huanan) AND (“severe acute respiratory” OR pneumonia) AND (outbreak)) OR “Coronavirus”[Mesh] OR “Coronavirus Infections”[Mesh] OR “COVID-19” [Supplementary Concept] OR “severe acute respiratory syndrome coronavirus 2” [Supplementary Concept] OR “Betacoronavirus”[Mesh] OR "COVID-19"[Mesh] OR "SARS-CoV-2"[Mesh]) AND (((pollut*[ti] OR qualit*[ti]) AND air[ti]) AND (air pollution[tiab] OR PM 2.5[tiab] OR PM5[tiab] OR PM10[tiab] OR Air pollutant*[tiab] OR particle*[tiab] OR particulate*[tiab] OR "Air Pollution"[Mesh] OR air quality[tiab] OR (air[ti] AND (quality*[ti] OR pollut*[ti])) OR air-quality[tiab] OR smog[tiab] OR soot[tiab] or gases[tiab] OR wood smoke[tiab] OR ozone[tiab] OR hydrocarbon*[tiab] OR polyaromatic hydrocarbon*[tiab] OR PAH[tiab] OR greenhouse gas*[tiab] OR carbon dioxide[tiab] OR methane[tiab] OR hydrofluorocarbon*[tiab] OR HFC[tiab] OR volatile organic compound*[tiab] OR VOC[tiab] OR CO2[tiab] OR combustion[tiab] OR emission*[tiab] OR Nitrogen oxide*[tiab] OR NOx[tiab] OR NO2[tiab] OR Sulphur dioxide[tiab] OR SO2[tiab] OR O3[tiab]))

EMBASE

No.	Query	Results
#3	#1 AND #2	
#2	(pollut*:ti OR qualit*:ti) AND air:ti AND ('air pollution'/exp OR 'pollution and pollution related phenomena'/exp OR 'particulate matter'/exp OR 'particulate matter 2 5'/exp OR 'particulate matter 10'/exp OR 'airborne particle'/exp OR 'air quality'/exp OR 'wood smoke'/exp OR 'air pollutant'/exp OR 'air pollution':ab,ti OR 'pm 2.5':ab,ti OR pm5:ab,ti OR pm10:ab,ti OR 'air pollutant*':ab,ti OR particle*:ab,ti OR particulate*:ab,ti OR (air:ti AND (quality*:ti OR pollut*:ti)) OR 'air quality':ab,ti OR smog:ab,ti OR soot:ab,ti OR gases:ab,ti OR 'wood smoke':ab,ti OR ozone:ab,ti OR hydrocarbon*:ab,ti OR 'polyaromatic hydrocarbon*':ab,ti OR pah:ab,ti OR 'greenhouse gas*':ab,ti OR 'carbon dioxide':ab,ti OR methane:ab,ti OR hydrofluorocarbon*:ab,ti OR hfc:ab,ti OR 'volatile organic compound*':ab,ti OR voc:ab,ti OR co2:ab,ti OR combustion:ab,ti OR emission*:ab,ti OR 'nitrogen oxide*':ab,ti OR nox:ab,ti OR no2:ab,ti OR 'sulphur dioxide':ab,ti OR so2:ab,ti OR o3:ab,ti)	26888
#1	(coronavirus OR ‘corona virus’ OR coronavirinae OR coronaviridae OR betacoronavirus OR covid19 OR ‘covid 19’ OR nCoV OR ‘CoV 2’ OR CoV2 OR sarscov2 OR 2019nCoV OR ‘novel CoV’ OR ‘wuhan virus’ OR novel coronavirus:ab,ti OR 2019-ncov:ab,ti OR coronavirus:ab,ti OR coronavirus disease-9:ab,ti OR coronavirus*:ab,ti OR coronovirus*:ab,ti OR 2019-nCoV:ab,ti OR COVID-19:ab,ti OR Covid:ab,ti OR ncov:ab,ti OR SARS-CoV-2:ab,ti OR SARSCov19:ab,ti OR SARS-Covid:ab,ti OR ((wuhan OR hubei OR huanan) AND (‘severe acute respiratory’ OR pneumonia) AND (outbreak)) OR 'coronavirus disease 2019'/exp OR 'Coronavirus infection'/exp OR 'Coronavirinae'/exp OR 'Betacoronavirus'/exp)	

Downloaded from www.annualreviews.org. Guest (guest) IP: 128.36.7.150 On: Sat, 09 Nov 2024 21:26:49

Supplemental Material: Annu. Rev. Public Health 2023. 44:1-20
https://doi.org/10.1146/annurev-publhealth-071521-120424
A Literature Review of the Effects of Air Pollution on COVID-19 Health Outcomes Worldwide: Statistical Challenges and Data Visualization
Bhaskar et al.

WEB OF SCIENCE
TIMESPAN: ALL YEARS. DATABASES: WOS, BCI, BIOSIS, CABI, CCC, DRCI, DIIDW, KJD, MEDLINE, RSCI, SCIELO, ZOOREC.

TOPIC: ("novel coronavirus" OR 2019-ncov OR coronavirus OR "coronavirus disease-9" OR coronavirus* OR coronovirus* OR "2019-nCoV" OR COVID-19 OR Covid OR ncov OR SARS-CoV-2 OR SARSCov19 OR "SARS-Covid" OR "covid 19" OR "Coronaviridae" OR "Coronaviridae infection" OR "coronavirus disease 2019") AND TOPIC: ((((pollut* OR qualit*) AND air) AND ("air pollution" OR "pollution and pollution related phenomena" OR "particulate matter" OR "particulate matter 2 5" OR "particulate matter 10" OR "airborne particle" OR "air quality" OR "wood smoke" OR "air pollutant" OR "air pollution" OR "PM 2.5" OR PM5 OR PM10 OR "Air pollutant*" OR particle* OR particulate* OR "air quality" OR (air AND (quality* OR pollut*)) OR air-quality OR smog OR soot OR gases OR "wood smoke" OR ozone OR hydrocarbon* OR "polyaromatic hydrocarbon*" OR PAH OR "greenhouse gas*" OR "carbon dioxide" OR methane OR hydrofluorocarbon* OR HFC OR "volatile organic compound*" OR VOC OR CO2 OR combustion OR emission* OR "Nitrogen oxide*" OR NOx OR NO2 OR "Sulphur dioxide" OR SO2 OR O3)))

COCHRANE COVID-19 STUDY REGISTER
(Journal Articles)
Filtered by: pollution OR pollutant OR pollutants OR particulate OR particulates OR air quality OR PM5 OR PM10 OR volatile OR VOC

Update: Filtered by
pollution OR pollutant OR pollutants OR particulate OR particulates OR air quality OR PM5 OR PM10 OR volatile OR VOC

MEDRXIV and **BIORXIV**
A reminder: these are preliminary reports that have not been peer-reviewed. They should not be regarded as conclusive, guide clinical practice/health-related behavior, or be reported in news media as established information. Not included in review
for term "pollution OR pollutant OR pollutants OR particulate OR particulates OR air quality OR PM5 OR PM10 OR volatile OR VOC AND Covid"

Downloaded from www.annualreviews.org. Guest (guest) IP: 128.36.7.150 On: Sat, 09 Nov 2024 21:26:49
